# Coordinate Regulation of Stem Cell Competition by Slit-Robo and JAK-STAT Signaling in the *Drosophila* Testis

**DOI:** 10.1371/journal.pgen.1004713

**Published:** 2014-11-06

**Authors:** Rachel R. Stine, Leah J. Greenspan, Kapil V. Ramachandran, Erika L. Matunis

**Affiliations:** Department of Cell Biology, Johns Hopkins University School of Medicine, Baltimore, Maryland, United States of America; The University of Texas Southwestern Medical Center, United States of America

## Abstract

Stem cells in tissues reside in and receive signals from local microenvironments called niches. Understanding how multiple signals within niches integrate to control stem cell function is challenging. The *Drosophila* testis stem cell niche consists of somatic hub cells that maintain both germline stem cells and somatic cyst stem cells (CySCs). Here, we show a role for the axon guidance pathway Slit-Roundabout (Robo) in the testis niche. The ligand Slit is expressed specifically in hub cells while its receptor, Roundabout 2 (Robo2), is required in CySCs in order for them to compete for occupancy in the niche. CySCs also require the Slit-Robo effector Abelson tyrosine kinase (Abl) to prevent over-adhesion of CySCs to the niche, and CySCs mutant for *Abl* outcompete wild type CySCs for niche occupancy. Both Robo2 and Abl phenotypes can be rescued through modulation of adherens junction components, suggesting that the two work together to balance CySC adhesion levels. Interestingly, expression of *Robo2* requires JAK-STAT signaling, an important maintenance pathway for both germline and cyst stem cells in the testis. Our work indicates that Slit-Robo signaling affects stem cell function downstream of the JAK-STAT pathway by controlling the ability of stem cells to compete for occupancy in their niche.

## Author Summary

Stem cells adhere to niches, or local microenvironments, which provide essential maintenance cues. In the *Drosophila* testis niche, quiescent hub cells maintain adjacent germline and somatic stem cells (or cyst stem cells, CySCs) via local JAK-STAT signaling. Here, we show that the Slit-Robo and JAK-STAT pathways integrate to modulate stem cell-niche cell adhesion. The ligand Slit is expressed in the hub, and Slit's receptor Robo2, which is transcriptionally activated by JAK-STAT signaling, is required in adjacent CySCs to promote ECad-based adhesion to the hub. Abl tyrosine kinase acts downstream of Robo2 to attenuate CySC-hub adhesion and prevent CySCs from outcompeting neighboring cells from the niche. Interestingly, Robo receptors mediate stem cell-niche adhesion during hematopoiesis, but the mechanisms are not understood. We suggest that the Slit-Robo-Abelson and JAK-STAT pathways may coordinately regulate stem cell-niche cell adhesion more generally.

## Introduction

**Figure 1:**
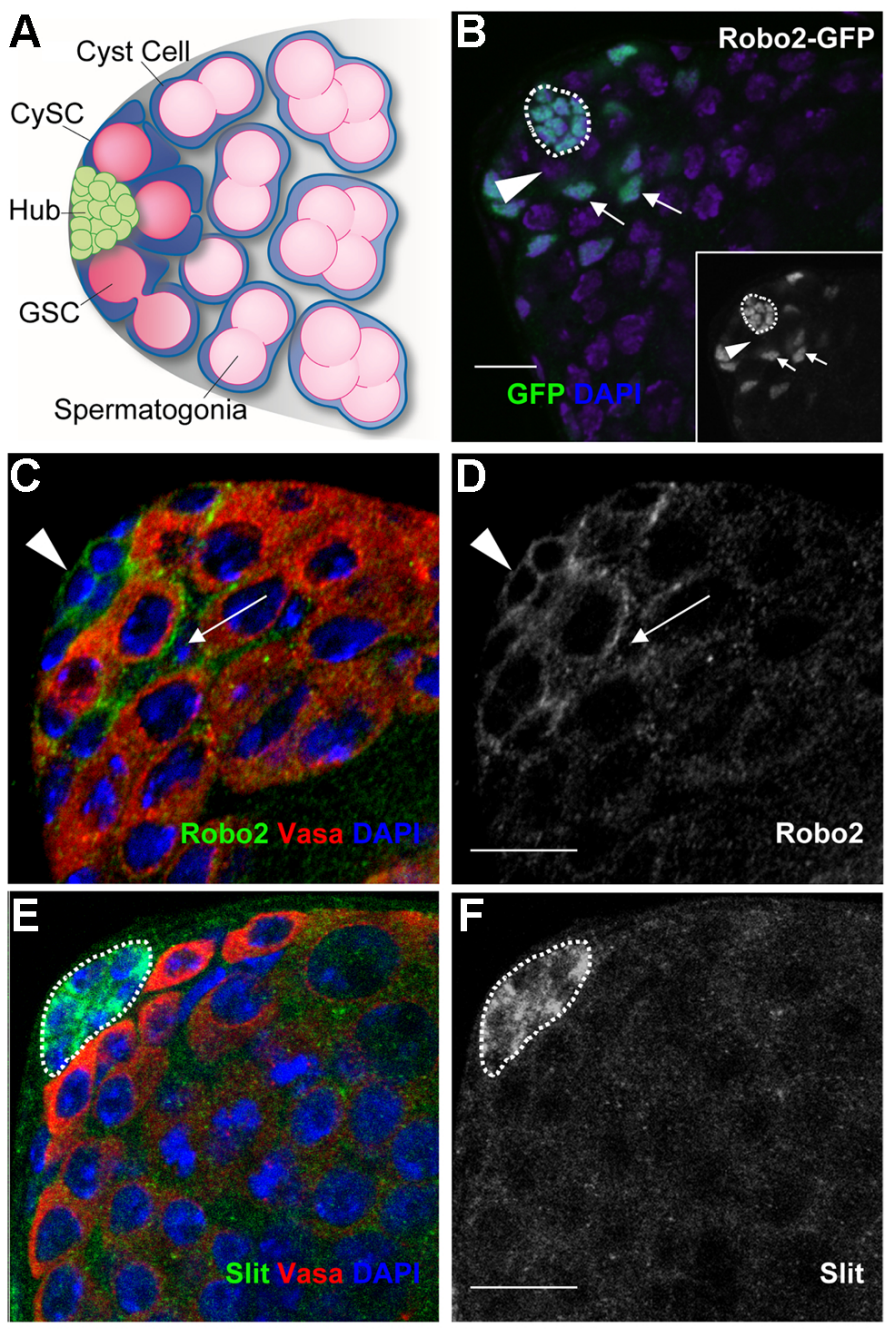
Components of the Slit-Robo pathway are expressed in the *Drosophila* testis stem cell niche. (A) The *Drosophila* testis apex. Germline stem cells (GSCs) and somatic cyst stem cells (CySCs) contact the hub. GSCs divide asymmetrically to give differentiating daughters that undergo four rounds of mitosis, producing interconnected spermatogonia that will eventually form sperm. CySCs divide to give non-mitotic somatic cyst cells that encase and support differentiating spermatogonia. (B) Confocal image of a testis apex containing a GFP enhancer trap that reports the expression of *robo2*. GFP (green) is expressed in the hub (outlined), CySCs and early cyst cell daughters (arrows), but not in GSCs (arrowhead) or their progeny. Inset is GFP channel alone. (C and E) Confocal sections of wild type testes with Vasa staining the germline (red). (C) Robo2 protein (green) is enriched at the cell surface around hub cells (arrowhead) and CySCs (arrow). (D) Robo2 alone (green channel). (E) Slit protein (green) is enriched in hub cells (outlined). (F) Slit alone (green channel). DNA stained with DAPI (blue), scale bars = 10 µm.

Adult stem cells are essential for tissue regeneration and are maintained in specialized microenvironments, or niches. Niches consist of the cells and extracellular structures required to support a specific stem cell population [1]. Signals produced by niches maintain stem cells by concomitantly repressing differentiation and promoting stem cell adhesion to the niche. Although many extracellular signals and intrinsic adhesion factors are known to be required for stem cell maintenance, little is known about how they converge to regulate stem cell-niche cell adhesion in vivo [2]. We have approached this question using the well-characterized niche within the *Drosophila* testis. In this tissue, a cluster of quiescent, somatic hub cells contributes to the stem cell niche by signaling to adjacent germline and somatic stem cells (GSCs and cyst stem cells, or CySCs) (Figure 1A) [3]. Both stem cell populations adhere to the hub via E-cadherin (ECad)-mediated adherens junctions [2], [4]. GSC divisions are stereotypically oriented such that, following mitosis, one daughter remains within the niche (and remains a GSC), while the other is displaced from the niche and typically enters the differentiation program [5]–[7]. CySCs also divide asymmetrically, and their differentiating progeny (cyst cells) encase differentiating germ cells and support their differentiation [8]–[10].

Multiple extrinsic signals act within this niche, the most well-understood being the JAK-STAT pathway, wherein local cytokine production from the hub activates STAT within both GSCs and CySCs to promote their maintenance [11], [12]. Currently only a few STAT targets acting in this niche are known; these include the putative transcriptional repressors Zfh-1 and Chinmo, which autonomously prevent CySC differentiation (or maintain CySC fate), but are dispensable within GSCs [3], [13], [14]. In contrast, the primary role of STAT within GSCs is to promote ECad-mediated adhesion to the hub, rather than to maintain GSC fate [15]. ECad is also required within CySCs for their maintenance, but its upstream regulators remain to be identified [4], [15].

Although stem cell-niche cell adhesion is an essential aspect of stem cell maintenance, it is becoming apparent that the modulation of adhesion levels in individual stem cells can affect their ability to compete for limited space and signals in a niche [16]. In the *Drosophila* ovary, ECad levels mediate stem cell competition between GSCs; this process is thought to serve as a quality control mechanism to eliminate less fit stem cells from the niche [17]. In the *Drosophila* testis, although it is not known whether ECad levels mediate stem cell competition, CySCs upregulating integrin gain a competitive advantage in the niche, outcompeting both GSCs and CySCs with lower integrin levels [18]. Since integrin-mediated adhesion is not intrinsically required within CySCs for their maintenance, the mechanisms linking niche signals and integrin-mediated adhesion within CySCs are not understood. In general, little is known about the coordination of multiple signals within niches, but such coordination is likely to be a fundamental aspect of niche biology that will modulate many aspects of stem cell behavior, including stem cell competition. Model systems like the *Drosophila* gonad, where a single stem cell and its progeny can be assayed over time, facilitate understanding stem cell competition in vivo, since a given mutation is thought to yield very different outcomes depending on whether it affects a few or all of the stem cells in question [19].

In this study, we discover a role for the Slit-Roundabout (Robo) pathway in the *Drosophila* testis stem cell niche. First identified for its role in axon guidance in the *Drosophila* central nervous system, Slit-Robo is a functionally conserved cell migration pathway [20]–[24]. In *Drosophila*, three Roundabout receptors (Robo, Robo2 [also called Leak] and Robo3) are activated by a single secreted ligand, Slit [25]. In addition to cell migration, Slit-Robo signaling can affect cell adhesion in both vertebrates and *Drosophila* [26]–[28], often in conjunction with the highly conserved cytoplasmic kinase and proto-oncogene Abelson (Abl tyrosine kinase in *Drosophila* and c-Abl in vertebrates) [26], [29]. Interestingly, Robo4 is required for adhesion of murine hematopoietic stem cells to their niches in the bone marrow, but the underlying mechanisms are not understood [30]. Though Abl has been studied extensively in the context of cancer, virtually nothing is known about the role(s) of Abl in endogenous stem cell niches. Here, we demonstrate that JAK-STAT responsive Slit-Robo-Abl signaling affects adhesion-mediated competition in adult stem cells.

## Results

### Slit-Robo pathway members are expressed in the testis stem cell niche

Since genes regulating stem cells often display expression patterns restricted to stem cells or their niches [3], we sought to identify new stem cell regulators by screening a collection of gene traps for patterns restricted to the *Drosophila* testis apex [31]. We found that an enhancer trap inserted in the *robo2* gene, encoding the Robo2 axon guidance receptor, is expressed in the hub, CySCs and their immediate daughters (Figure 1B). Immunostaining confirmed that Robo2 is enriched on the cell surface of hub cells and CySCs, as expected for a transmembrane receptor, but decreases in expression in cells further from the niche (Figure 1C, 1C′). Since GSCs are enveloped by CySCs and the two membranes are difficult to distinguish by confocal microscopy, it is unclear if GSCs also express Robo2. Nonetheless, the enrichment of Robo2 within CySCs suggests this receptor may relay signals to them. Accordingly, the secreted ligand Slit, which signals through Robo2 [20], is enriched specifically in hub cells in a punctate pattern, consistent with the expression pattern of other hub-secreted ligands (Figure 1D, 1D′) [11], [12], [32]. Together, these data suggest that Slit locally activates Robo2 in adjacent germline and/or cyst stem cells.

### Robo2 is required for CySC but not GSC maintenance

**Figure 2:**
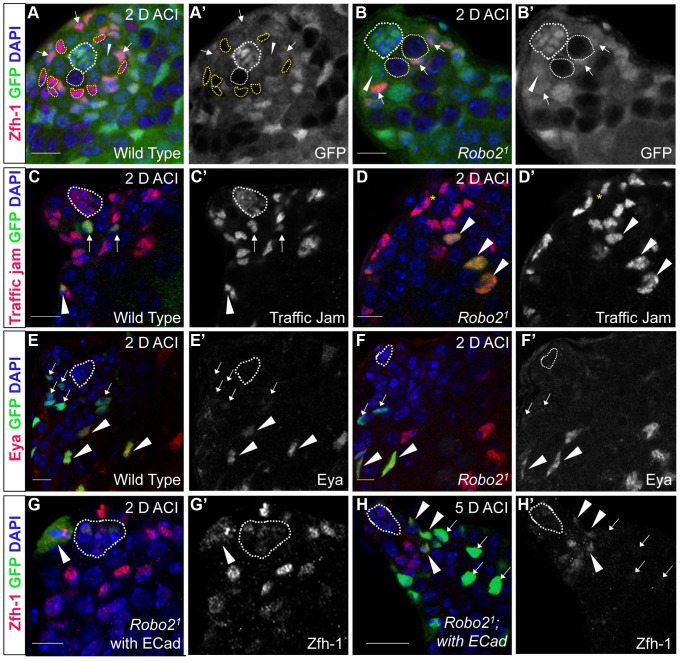
The Robo2 receptor is autonomously required for CySC maintenance in the testis. (A–B) Confocal sections of testes with Zfh-1 staining CySCs and early cyst cell daughters (red). Negatively marked mosaic clones are identified by absence of GFP (green). (A) At 2 days ACI, wild type CySC clones (outlined, yellow) and GSC clones (outlined, white) are both present in the testis while (B) *robo2^1^* mutant CySCs are absent at 2 days ACI and only GSC clones (outlined, white) are present. For comparison, examples of GFP^+^ GSCs (arrowheads) and CySCs (arrows) are indicated. A′ and B′ show GFP panels alone. (C–D) Confocal sections of testes with Traffic jam staining the hub, CySCs and cyst cells (red). Positively marked mosaic clones are identified by presence of GFP (green). (C) At 2 days ACI, marked wild type CySCs (arrows) are present close to the hub while differentiating cyst cells are present further from the hub. (D) *robo2^1^* mutant CySCs are absent near the hub but *robo2^1^* null differentiating cysts cells (arrowheads) are present far from the hub (yellow asterisk, below plane of focus). C′ and D′ show Traffic Jam staining alone. (E–F) Confocal sections of testes with Eya staining differentiating late cyst cells (red). Positively marked mosaic clones are identified by presence of GFP (green). (E) Eya^+^ wild type cyst cell clones (arrowheads) and (F) *robo2^1^* cyst cell clones (arrowheads) are present outside the niche at 2 days ACI. Early cyst cell clones (both wild type and *robo2^1^*) do not express Eya (arrows) E′ and F′ show Eya staining alone. (G–H) Confocal sections of testes with Zfh-1 staining CySCs and early cyst cell daughters (red). Positively marked mosaic clones are identified by presence of GFP (green). (G) At 2 days ACI, overexpression of ECad rescues *robo2* null clones, which are capable of dividing (arrowhead indicates mitotic GFP^+^ CySC, DNA condensed, and nuclear GFP and Zfh-1 proteins appear cytoplasmic). (H) *Robo2* null CySCs rescued with ECad (arrowhead) are maintained at 5 days ACI and produce progeny (arrows). G′ and H′ show Zfh-1 staining alone. Hubs outlined in white, DNA stained with DAPI (blue), scale bars = 10 µm.

Since Slit-Robo signaling is essential during development [33], we used mosaic analysis to generate a small number of *robo2*-null cells, called clones, in an otherwise normal adult testis. We used two alleles of *robo2* (robo2^1^ and robo2^8^), both of which carry a premature stop codon and are classified as null alleles [23]. At 2, 4, 8 and 12 days after clone induction (ACI), we scored for the presence of negatively marked *robo2* null CySCs, identified as GFP-negative cells adjacent to the hub expressing high levels of the CySC/early cyst cell marker Zfh-1 [14]. Negatively marked wild type control CySCs were present at all timepoints, as expected (Figure 2A, Table 1). However, negatively marked *robo2* null CySCs were almost completely absent by 2 days ACI (Figure 2B, Table 1, reported as testes with CySC clones/total testes). In contrast, negatively marked wild type control and *robo2* null GSCs were maintained in similar numbers at all timepoints (Figure 2A, 2B, Table S1). These data indicate that *robo2* is autonomously required for CySC but not GSC maintenance. To determine how *robo2* null CySCs were lost from the niche, we first looked for the presence of *robo2* null CySC progeny (cyst cells). Because negatively marked cyst cells are difficult to detect, we repeated our mosaic analysis using the repressible cell marker (MARCM) technique in order to create positively marked clones [34]. In addition to confirming our negative clone data that Robo2 CySCs are rapidly lost from the niche, MARCM clonal analysis allowed us to detect positively marked cyst cells lacking *robo2*. These cells were identified by their distance from the hub and expression of Traffic jam, which marks the hub and CySC lineage (Figure 2C, 2D). We observed *robo2* null cyst cells in 80% of testes (compared to 87% in wild type controls, p-value = .70, n>30), suggesting that *robo2* null CySCs are capable of differentiating. To determine if marked *robo2* null CySCs leave the niche due to premature differentiation, we stained for the expression of the cyst cell differentiation factor Eyes absent (Eya). Eya marks cyst cells associated with late spermatogonia and spermatocytes, but is not detected at earlier stages of spermatogenesis (Figure 2E) [35]. However, Eya can be detected in CySCs that are null for the self-renewal factor Zfh-1 before they exit the niche [14]. Thus, the presence of Eya-positive cells within the niche can indicate a premature loss of CySC identity. We observed that CySCs and early cyst cells lacking *robo2* did not express Eya (Figure 2F), indicating that they were not differentiating prematurely. In contrast, older cyst cells lacking *robo2* (those associated with late spermatogonia and spermatocytes) did express Eya (Figure 2E, 2F). This further supports the idea that *robo2*-null cyst cells can differentiate normally. However, we cannot rule out the possibility that Robo2 is required for Eya expression since Robo2 protein may persist longer in non-mitotic cyst cells than CySCs, which divide frequently. To further determine if Robo2 controls CySC identity, we ectopically expressed Robo2 outside of the stem cell niche. Ectopic expression of CySC self-renewal factors such as Zfh-1 in the CySC lineage is sufficient to cause overproliferation and accumulation of CySCs and GSCs outside of the niche [3], [13], [14], [36]. However, ectopic Robo2 expression in CySCs and cyst cells is not sufficient to produce an accumulation of stem cells outside the testis niche (Figure S1A, S1B). Global ectopic expression of the JAK-STAT ligand Unpaired (Upd, also called Outstretched) is also sufficient to cause overproliferation of CySCs and GSCs outside the niche [11], [12], but ectopic expression of the ligand Slit does not cause stem cell overproliferation. Instead, ectopic Slit produces a mild phenotype where CySCs and early cyst cells tend to aggregate (Figure S1C, S1D). Together, these data suggest that Slit-Robo signaling is not sufficient to maintain CySC identity outside of the niche.

**Table 1:** Robo2 is required cell-autonomously for CySC maintenance in the *Drosophila* testis.

Genotype	2 days ACI	4 days ACI	8 days ACI	12 days ACI
	**Testes with CySC Clones** a			
**Wild type clones**	10/23 (43.5)	11/22 (50.0)	6/21 (28.6)	1/25 (4.0)
**Robo2^1^ clones**	1/25 (4.0)**	0/23 (0.0)**	0/25 (0.0)**	0/23 (0.0)
**Robo2^8^ clones**	2/24 (8.3)**	1/24 (4.2)**	0/27 (0.0)**	0/25 (0.0)

a Testes with CySC clones = testes with GFP^−^, Zfh-1^+^ cells/total testes scored (percentage).

** = p<.01 vs Wild type clones.

ACI = After Clone Induction.

We next asked whether Slit-Robo signaling normally promotes CySC viability by using Terminal deoxynucleotidyl transferase dUTP nick end labeling (TUNEL) to quantify dying cells within testes containing *robo2* null clones. Since *robo2* null CySCs are lost rapidly, before the activation of the mosaic marking system can be detected, we induced high levels of clones 24 hours before TUNEL labeling, when we estimate that there is at least one *robo2* null CySC per testis. After quantifying the number of dying cells in the region of the testis occupied by stem cells (within ∼10 microns of the hub, Figure S2), we found no significant difference between testes containing *robo2* null clones or wild type control clones (.03 vs. .06 cells per testis, p-value = 0.39, n>50 testes) (Figure S2A, S2B). As expected, TUNEL-positive spermatogonial cysts were present in the majority of both wild type and *robo2* mosaic testes [37], [38], confirming that our TUNEL-staining was effective. These data suggest that *robo2* is not required for CySC viability.

We then suspected that Robo2 might affect the ability of CySCs to compete for occupancy in the niche. To test this, we used the C587-gal4 driver to knock down *robo2* in CySCs and cyst cells [4]. Using the temperature-inducible Gal80^TS^ system, we initiated knockdown of *robo2* for 7 days in adult flies (0–5 days post eclosion). We found that, in contrast to our mosaic analysis results where *robo2*-null CySCs are rapidly lost from the niche, RNAi-induced knockdown of *robo2* in all CySCs and cyst cells in the adult testis via RNAi does not result in total CySC loss (Figure S3A, n = 25). Instead, testes appear grossly normal, with both GSCs and CySCs still present in the niche. This indicates that CySCs lacking Robo2 are competent to remain in the niche, but cannot compete for niche occupancy when more fit, wild type CySCs are present, although an alternative explanation for this result is that RNAi knockdown of *robo2* was partial compared to complete deletion of *robo2* in mosaic clones. We used the previously described *robo2* developmental testis phenotype as a method to confirm the effectiveness of our RNAi construct. We used C587-gal4 without Gal80^TS^ to initiate knockdown of *robo2* during development and scored for testis malformation as previously described [39]. Robo2 deletion is known to cause defects in the proper establishment of the embryonic stem cell niche with ∼30% penetrance [39]. When C587-gal4, which is expressed in the somatic gonadal precursors of the developing embryonic gonad [40], was used to drive RNAi mediated knockdown of *robo2*, 33% of testes appeared grossly abnormal. Consistent with published data, these testes exhibited decreased numbers of GSCs and hub cells as well as germline cells that appeared to be defective in normal differentiation (Figure S3B, n = 27). Taken together, our clonal analysis and adult RNAi knockdown experiments indicate that loss of Robo2 decreases the ability of CySCs to compete for their niche. Although CySCs lacking Robo2 are still maintained and functional, they cannot compete against wild type CySCs for occupancy and are quickly expelled from the niche when more fit wild type CySCs are present. We hypothesize that Robo2 maintains CySCs by promoting CySC-hub cell adhesion. Changes in stem cell adhesion are thought to modulate stem cell competition within niches more generally [17], [18], but the regulation of competition by niche signals is still not completely understood.

### Robo2 null CySCs are rescued by overexpression of E-cadherin

In both vertebrates and invertebrates, Slit-Robo signaling can modulate cell-cell adhesion by acting on cadherins including N-cadherin (Ncad) and E-cadherin (ECad) during development [26], [28]. Interestingly, both cadherins are highly enriched on the surface of hub cells (Figure S4) [5], [41]. However, mosaic analysis of two previously characterized loss-of-function *Ncad* alleles revealed that *Ncad* is not required for CySC maintenance (Table S2). In contrast, ECad is required clonally for CySC maintenance [4], but its regulation via niche signals is not understood. Therefore, we hypothesized that Slit-Robo signaling promotes ECad-mediated CySC-hub cell adhesion. To test this, we overexpressed ECad in *robo2* null CySC clones and found that this was sufficient to partially rescue these stem cells; rescued cells appeared morphologically similar to wild type CySC clones, and they were capable of proliferating and producing differentiating progeny (Figure 2G, 2H, Table 2). Although rescue of *robo2* mutant clones by ECad overexpression suggests that Robo2 promotes ECad-mediated adhesion of CySCs to the hub, we cannot rule out that increasing ECad expression could facilitate the rescue of CySCs indiscriminately, simply by making them “stickier”. However, previous publications have shown that overexpression of ECad is not able to rescue stem cells lacking a self-renewal factor [42]. Notably, if ECad is clonally knocked out of CySCs in the niche, ECad mutant CySCs are rapidly lost [4], but knockdown of ECad in all CySCs does not result in complete CySC loss (Figure S5). This indicates that ECad, like Robo2, is modulating CySC competition through adhesion. ECad is likely required for cell fitness and cells with lower ECad levels are eliminated from the niche. While we cannot rule out that ECad and Robo2 work in parallel, they both appear to modulate stem cell competition in a similar manner. Therefore, we sought to identify additional factors acting together with Robo2 and ECad that regulate stem cell-niche cell adhesion.

**Table 2:** Ecad overexpression or Abl knockdown partially rescues loss of Robo2 mutant CySC clones.

Genotype	2 days ACI		6 days ACI	
	Testes with CySC clones^a^	Testes with >50% marked CySCs^b^	Testes with CySC clones	Testes with >50% marked CySCs
**Wild type clones**	25/48 (52.1)	0/48 (0.0)	20/46 (43.5)	3/46 (6.5)
**Robo2^1^ clones**	2/30 (6.7)	0/30 (0.0)	0/31 (0.0)	0/31 (0.0)
**Wild type clones overexpressing Ecad** c	14/30 (46.7)	2/30 (6.7)	17/33 (51.5)	10/33 (30.3)* ^, ^ h
**Robo2^1^ clones overexpressing Ecad** d	20/69 (29.0)* ^, ^ g	0/69 (0.0)	5/26 (19.2)* ^,^ g	0/26 (0.0)
**Wildtype clones expressing Abl RNAi** e	13/22 (59.1)	1/22 (4.5)	14/21 (66.7)	9/21 (42.8)** ^, ^ h
**Robo2^1^ clones expressing Abl RNAi** f	11/23 (47.8)** ^, ^ g	0/23 (0.0)	ND	ND

a Testes with CySC clones = testes with GFP^+^, Zfh-1^+^ cells/total testes scored (percentage).

b Testes with >50% marked CySCs = testis where GFP^+^, Zfh-1^+^ cells>GFP^−^, Zfh-1^+^ cells/total testes scores (percentage).

c+e Wild type clones expressing Ecad or Abl-RNAi = wild type MARCM Frt 40A flies driving (c) Ecad or (e) Abl-RNAi specifically in induced clones.

d+f Robo2^1^ clones expressing Ecad or Abl-RNAi = Robo2^1^ MARCM flies driving (c) Ecad or (e) Abl-RNAi specifically in induced clones.

g P value compared to Robo2^1^ clones.

h P value compared to wild type clones.

ND = Not Determined, ACI = After Clone Induction,

* = P value<.05,

** = P value<.01.

### Abl-deficient CySCs outcompete wild type CySCs for niche occupancy

**Figure 3:**
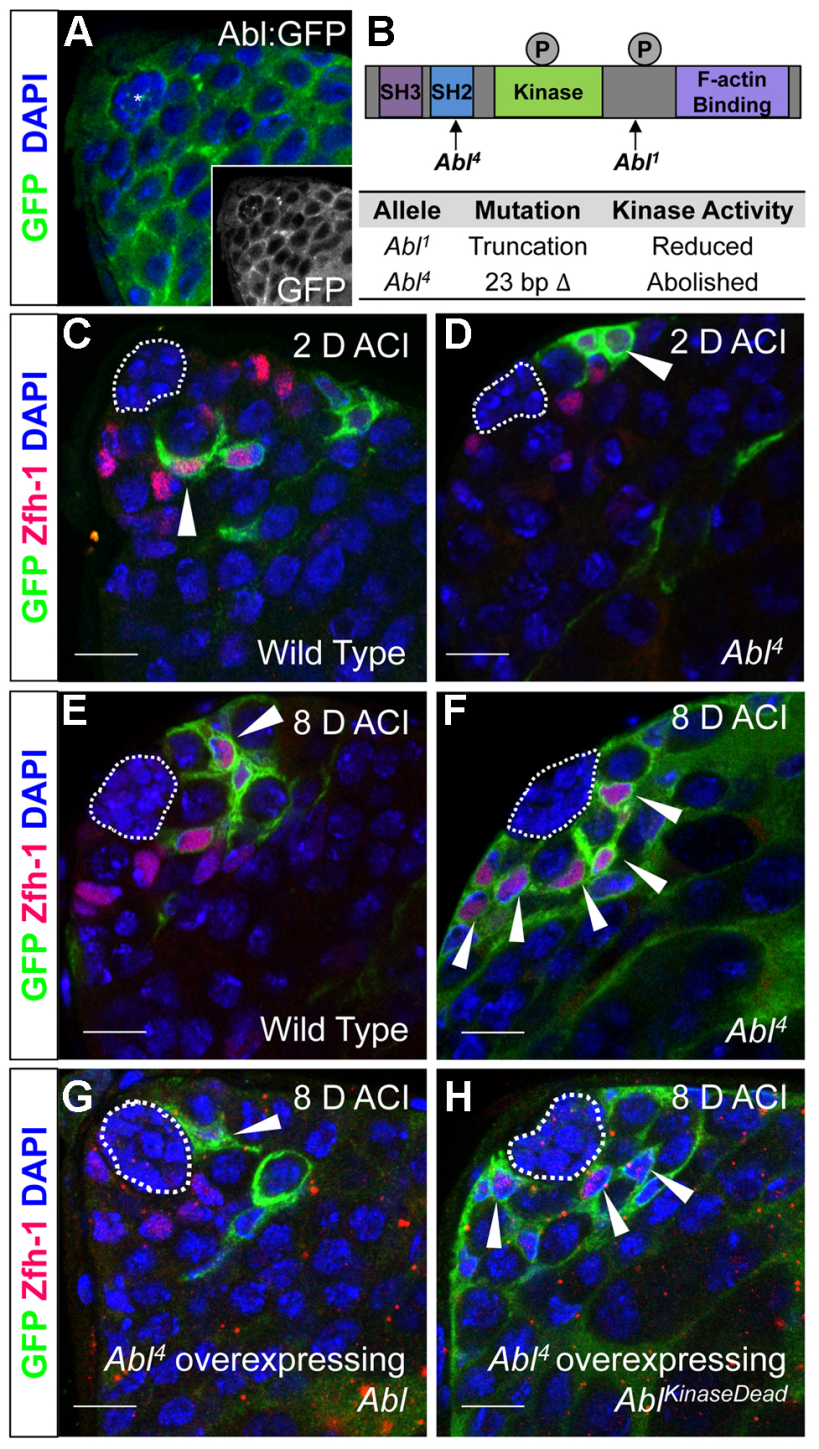
Abl kinase activity is required to prevent CySC overcompetition. (A) Confocal section of testis expressing an Abl∶GFP fusion protein. Abl is expressed in both germline and somatic lineages and is enriched at cell surfaces. The position of the hub is marked with an asterisk. (B) Structure of the Abl kinase protein with two *Abl* alleles described. (C–H) Confocal sections of testes with Zfh-1 staining CySCs and early cyst cell daughters (red). Positively marked mosaic clones are identified by presence of GFP (green). At 2 days ACI, (C) wild type CySC clones (arrowhead) are detected in similar numbers to (D) *Abl^4^* mutant CySC clones (arrowhead). At 8 days ACI, (E) the number of marked wild type CySCs per testis remains low while (F) the number of marked *Abl^4^* mutant CySCs per testis increases and few unmarked CySCs are detected. (G) At 8 days ACI, the number of marked *Abl^4^* mutant CySCs per testis does not increase compared to wild type when Abl is resupplied while (H) Abl^KinaseDead^ expression fails to rescue the increase in marked *Abl^4^* mutant CySCs per testis. Hubs outlined in white, DNA stained with DAPI (Blue), scale bars = 10 µm.

Although Slit-Robo signaling has been studied extensively during development, particularly in the nervous system, Abl kinase is one of the few known downstream effectors in this signaling pathway. Abl physically and genetically interacts with Robo in the *Drosophila* CNS [29], but less is known about Abl's interactions with the Robo paralog Robo2 [43]. Abl transcripts are enriched in the testis apex according to RNA-Seq analysis (Table S3) [44]. In addition, an Abl∶GFP fusion protein driven by the *Abl* promoter [45] is detected throughout the cytoplasm of all cells within the testis apex and with a slight enrichment at cell membranes (Figure 3A). This expression data is consistent with a role for Abl in stem cell-niche cell adhesion.

To determine whether Abl kinase is required for CySC maintenance, we used the MARCM technique to generate mosaic testes using the *Abl^4^* allele, which encodes a protein with a catalytically inactive kinase domain (Figure 3B) [46]. At 2 days ACI, wild type and *Abl^4^* marked CySCs were morphologically indistinguishable, and were present at similar frequencies (Figure 3C, 3D, Table 3). As expected, by 8 days ACI, the percentage of testes containing marked wild type CySCs decreased due to natural turnover. In contrast, the percentage of testes with marked *Abl^4^* CySCs remained high, indicating that CySCs lacking Abl kinase activity are maintained better than wild type CySCs (Table 3). In addition, by 8 days ACI the number of *Abl^4^* CySCs per testis increased significantly compared to wild type CySCs (12.1 versus 3.8 marked CySCs per testis, p<.0001, n>16; Figure 3E, 3F). Since the total number of CySCs did not increase in testes containing *Abl^4^* CySC clones compared to controls (18.2 CySCs per testis vs. 20.0 CySCs testis, p = .0144), we conclude *Abl^4^* mutant CySCs (or their progeny) are capable of displacing, or outcompeting, neighboring wild type CySCs from the niche. Similarly, clones of CySCs expressing Abl RNAi also displayed increased competition for niche occupancy (Figure S6A–B, Table 2).

**Table 3:** Abl kinase activity is required to attenuate CySC competition in the *Drosophila* testis.

Genotype	2 days ACI		6 days ACI		8 days ACI		10 days ACI	
	Testes with CySC clones^a^	Testes with >50% marked CySCs^b^	Testes with CySC clones	Testes with >50% marked CySCs	Testes with CySC clones	Testes with >50% marked CySCs	Testes with CySC clones	Testes with >50% marked CySCs
**Wild type clones**	25/34 (73.5)	1/34 (2.9)	13/29 (44.8)	0/29 (0.0)	14/31 (45.1)	0/31 (0.0)	7/32 (21.9)	1/32 (3.1)
**Abl^1^ clones**	16/26 (61.5)	0/26 (0.0)	7/17 (41.2)	3/17 (17.6)	11/25 (44.0)	3/25 (12.0)*	9/25 (36.0)	4/25 (16.0)
**Abl^4^ clones**	14/22 (63.6)	1/22 (4.5)	24/45 (53.3)	12/45 (26.7)	15/26 (57.7)	11/26 (42.3)***	14/28 (50.0)***	12/28 (42.8)***
**Abl^4^ clones expressing Abl** c	5/18 (27.8)	0/18 (0.0)	ND	ND	4/21 (19.0)	0/21 (0.0)	ND	ND
**Abl^4^ clones expressing Abl^KD,^** d	6/9 (66.7)	1/9 (11.1)	ND	ND	11/27 (40.7)	8/27 (29.6)***	ND	ND

a Testes with CySC clones = testes with GFP^+^, Zfh-1^+^ cells/total testes scored (percentage).

b Testes with >50% marked CySCs = testis where GFP^+^, Zfh-1^+^ cells>GFP^−^, Zfh-1^+^ cells/total testes scores (percentage).

c Abl^4^ MARCM clones expressing Abl = Abl^4^ MARCM flies driving UAS-Abl specifically in induced clones.

d Abl^4^ MARCM clones expressing Abl^KinaseDead^ = Abl^4^ MARCM flies driving UAS-Abl^KinaseDead^ specifically in induced clones.

ND = Not Determined, ACI = After Clone Induction,

* = p value<.05 vs Wild type clones,

*** = p value<.001 vs Wild type clones.

After confirming that *Abl^4^* mutant CySCs could outcompete their wild type neighbors, we sought to characterize how Abl was controlling CySC competition. We first asked whether the CySC competition that we observed was dependent on the kinase activity of Abl. Although Abl phosphorylates many targets, it also has kinase-independent functions [47], [48]. However, only wild type Abl, but not kinase-dead Abl, was sufficient to rescue *Abl^4^* (kinase dead) clones (Figure 3G, 3H, Table 3). Furthermore, CySC clones of the *Abl^1^* allele, which encodes a truncated form of Abl that retains residual kinase activity (Figure 3B) [46], were only weakly capable of outcompeting their wild type CySC neighbors (Table 3). Together, these data indicate that Abl kinase activity is required to prevent CySCs from outcompeting neighboring CySCs from the niche. Additionally, since the *Drosophila* testis niche contains two distinct populations of stem cells, we wondered whether mutant CySCs could also outcompete their GSC neighbors. In the *Drosophila* testis, *Socs36E* mutant CySC clones are known to outcompete not only neighboring CySCs but also GSCs through an integrin-dependent mechanism [18]. However, we found that testes containing *Abl^4^* mutant clones had a normal complement of GSCs (8.0 GSCs per testis at 8 days ACI) indicating that *Abl^4^* mutant CySC clones could not outcompete their GSC neighbors. This suggests that Abl and Socs36E act on cell competition using distinct mechanisms.

Although most cases of stem cell competition in a niche are linked to increased adhesion, there are examples in both mammals and *Drosophila* of increased levels of proliferation causing stem cells to take over a niche [16], [49], [50]. Abl activity is known to promote cell proliferation in some cancers [51], but less is known about these effects in non-cancerous tissues. To test if cell proliferation rates were affected in *Abl^4^* mutant CySCs, we calculated the mitotic index by staining testes containing *Abl^4^* clones with the mitotic marker phospho-histone H3. We compared the mitotic index of marked *Abl^4^* homozygous CySCs to that of neighboring heterozygous CySCs at 6 days ACI (when about half of the CySCs in each testis are mutant), but we found no significant difference between the mitotic indices of *Abl^4^* CySCs and their neighbors (.0077 vs .025 mitotic CySCs/total CySCs, n>194 cells, p value = .125). If anything, Abl mutant CySCs divide less frequently than their neighbors, although this difference was not statistically significant. This suggests that increased adhesion rather than proliferation is most likely the cause of Abl-mediated cell competition in the testis niche. This idea is consistent with previous findings that increased cell-cell adhesion is sufficient to promote stem cell competition without altering stem cell division rates [17], [18], and it suggests that Abl kinase activity may modulate CySC competition by attenuating adhesion within CySCs.

### E-cadherin is required for Abl mediated cell competition in the testis

**Figure 4:**
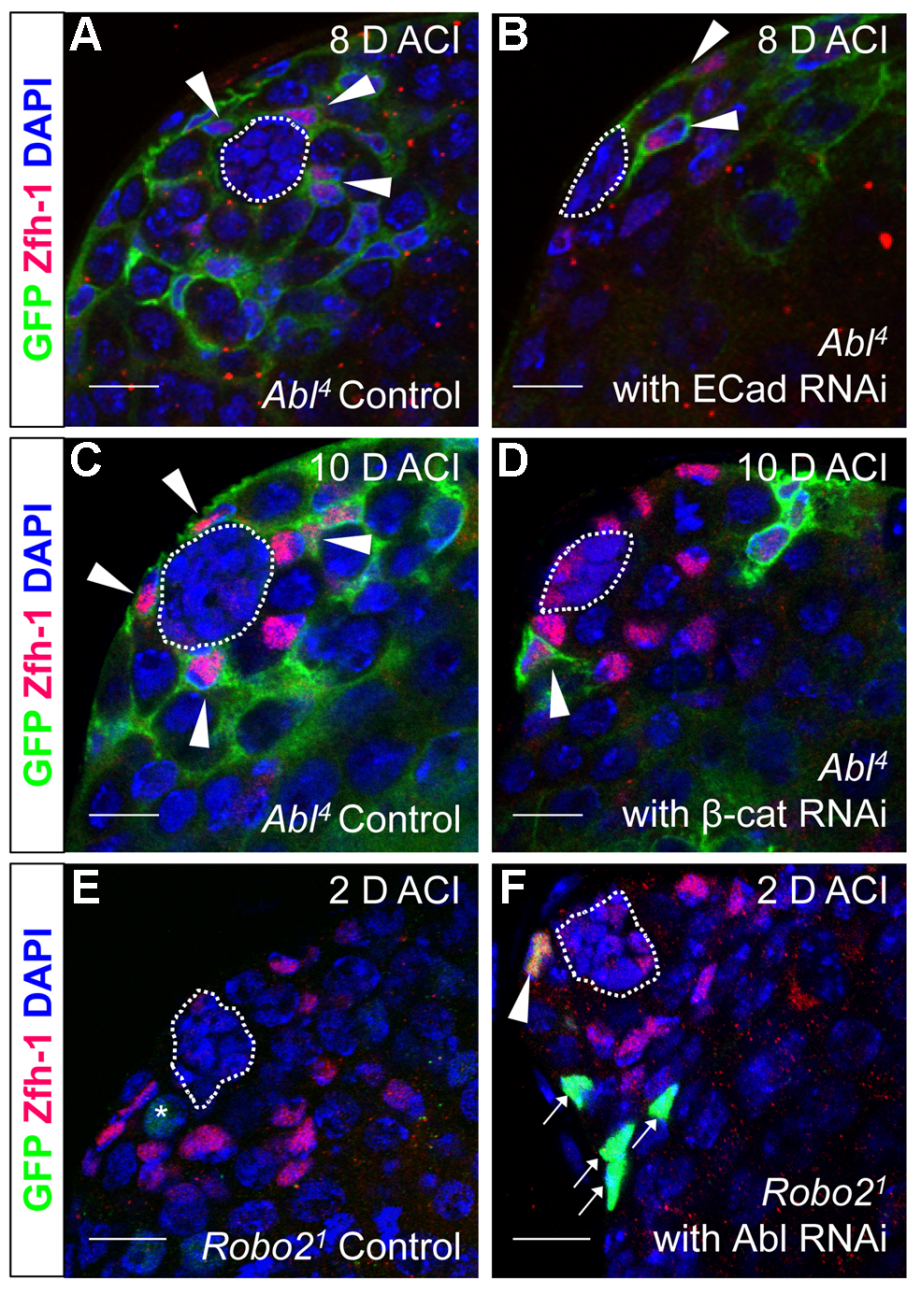
Robo2 and Abl alter cell-cell adhesion to control CySC maintenance. (A–F) Confocal sections of testes with Zfh-1 staining CySCs and early cyst cell daughters (red). Positively marked mosaic clones are identified by presence of GFP (green). At 8 days ACI (A) control *Abl^4^* CySCs (arrowheads) are present at high numbers in each testis, while (B) the number of *Abl^4^* CySCs expressing ECad RNAi (arrowhead) remains low. At 10 days ACI, (C) marked *Abl^4^* CySCs (arrowheads) are present in high numbers per testis, while (D) the number of *Abl^4^* CySCs expressing β-cat RNAi (arrowhead) remains low. At 2 days ACI, (E) *robo2* null GSCs (asterisk), but not CySCs are present while (F) *robo2* null CySC expressing Abl RNAi (arrowhead) are present in the niche and produce cyst cell daughter cells (arrows). Hubs outlined in white, DNA stained with DAPI (blue), scale bars = 10 µm.

As mentioned above, testes with *Abl* mutant CySC clones retain a full complement of GSCs, while testes with *Socs36E* mutant CySCs do not [18], indicating that Abl and Socs36E may control stem cell competition in different ways. Abl is known to affect adhesion levels in multiple tissues [26], [48], [52], [53], usually by destabilizing adherens junctions. Therefore, we focused on cadherins, rather than integrins as a potential mechanism for adhesion-based stem cell competition in Abl mutant CySCs. We first hypothesized that ECad mediated adhesion is normally attenuated by Abl kinase in CySCs. If this is true, reducing ECad in CySCs lacking Abl kinase activity should eliminate their competitive advantage. We induced control (*Abl^4^*) and experimental (*Abl^4^* with ECad RNAi) CySC clones at similar frequencies, but by 8 days ACI, the latter clones did not outcompete their neighbors. Instead, they were present in similar numbers as wild type CySCs (Figure 4A, 4B, Table 4). This indicates that Abl mutant CySCs require ECad to outcompete their neighbors for occupancy in the niche. While Abl and ECad could be working in parallel pathways, we suspect that Abl normally destabilizes ECad adherens junctions to mediate CySC competition. In this case, knocking down ECad would cause *Abl*-null CySCs to lose their competitive advantage compared to neighboring wild type cells. Because ECad RNAi is not completely efficient at knocking down ECad levels (predicted efficiency, 48.7–50.3%, [54]), the residual ECad would still be stabilized in Abl mutant CySCs, leading to an intermediate phenotype where cells are better maintained than in clones expressing ECad RNAi alone. This is what we observe and our data suggests that Abl-mediated competition relies on increased ECad-mediated adhesion to the hub. Consistent with this idea, ectopic expression of ECad within individual CySCs was sufficient to cause them to outcompete neighboring wild type CySCs but not GSCs from the niche (Figure S6C, Table 2). Taken together, these data suggest that Abl kinase activity destabilizes ECad-mediated adherens junction complexes in CySCs to attenuate CySC-hub cell adhesion. However, as we have not shown a direct interaction between Abl and ECad in the testis niche, it is possible that these molecules act in parallel.

**Table 4:** Ecad knockdown prevents Abl^4^ mutant CySC clones from outcompeting their neighbors and taking over the niche.

Genotype	2 days ACI		6 days ACI		8 days ACI	
	Testes with CySC clones^a^	Testes with >50% marked CySCs^b^	Testes with CySC clones	Testes with >50% marked CySCs	Testes with CySC clones	Testes with >50% marked CySCs
**Wild type Control**	8/16 (50.0)	0/16 (0.0)	6/16 (37.5)	1/16 (6.25)	ND	ND
**Abl^4^ Clones**	9/14 (64.3)	0/14 (0.0)	ND	ND	14/19 (73.7)	11/19 (57.9)
**Wild type clones expressing Ecad RNAi (8024)** c	15/20 (75.0)	0/20 (0.0)	3/20 (15.0)	0/20 (0.0)	ND	ND
**Abl^4^ clones expressing Ecad RNAi (8024)** d	9/14 (64.3)	0/14 (0.0)	ND	ND	6/18 (33.3)	2/18 (11.1)** ^, ^ g
**Wild type clones expressing Ecad RNAi (27081)** e	11/25 (44.0)	0/25 (0.0)	2/16 (12.5)	0/16 (0.0)	ND	ND
**Abl^4^ clones expressing Ecad RNAi (27081)** f	13/25 (52.0)	0/25 (0.0)	ND	ND	7/15 (46.7)	3/15 (20.0)** ^, ^ g

a Testes with CySC clones = testes with GFP^+^, Zfh-1^+^ cells/total testes scored (percentage).

b Testes with >50% marked CySCs = testis where GFP^+^, Zfh-1^+^ cells>GFP^−^, Zfh-1^+^ cells/total testes scores (percentage).

c+e Wild type clones expressing Ecad RNAi = Abl^4^ MARCM flies driving (c) Ecad RNAi (VDRC8024) or (e) Ecad RNAi (VDRC27081) specifically in induced clones.

d+f Abl^4^ clones expressing Ecad RNAi = Abl^4^ MARCM flies driving (c) Ecad RNAi (VDRC8024) or (e) Ecad RNAi (VDRC27081) specifically in induced clones.

g P value vs Abl^4^ clones.

ND = Not Determined, ACI = After Clone Induction,

* = P value<.05,

** = P value<.01.

### β-catenin is required for Abl-mediated stem cell competition in the testis

In both *Drosophila* and vertebrates, Abl can decrease cell-cell adhesion by phosphorylating β-catenin (β-cat) to destabilize adherens junctions [52]. In the testis apex, the *Drosophila* β-cat ortholog Armadillo is highly enriched at the surface of hub cells (Figure S4C) [5]. We hypothesized that β-cat is phosphorylated and destabilized by Abl kinase, leading to attenuation of ECad-mediated adhesion in CySCs. If so, reducing β-cat in CySCs lacking Abl kinase should decrease the ability of CySCs to outcompete their neighbors from the niche. We tested this hypothesis by expressing β-cat-RNAi in *Abl^4^* clones using MARCM, and assaying for the ability of marked mutant CySCs to outcompete their wild type neighbors. At 2 days ACI, control (*Abl^4^*) and experimental (*Abl^4^* with β-cat-RNAi) clones were induced at similar frequencies. However, by 10 days ACI, experimental clones did not outcompete their neighbors; instead they displayed similar kinetics to wild type CySC clones (Figure 4C, 4D, Table 5). While β-cat could be modulating cell adhesion in a parallel pathway to Abl, we can conclude that *Abl^4^* mutant CySCs require β-cat to outcompete their neighbors. Given the literature supporting an interaction between Abl and β-cat [26], we suggest a model in which Abl attenuates CySC-hub cell adhesion by phosphorylating β-cat to destabilize adherens junction complexes.

**Table 5:** β-Cat knockdown prevents Abl^4^ mutant CySC clones from outcompeting their neighbors and taking over the niche.

Genotype	2 Days ACI		6 days ACI		10 days ACI	
	Testes with CySC clones^a^	Testes with >50% marked CySCs^b^	Testes with CySC clones	Testes with >50% marked CySCs	Testes with CySC clones	Testes with >50% marked CySCs
**Wild type clones**	8/16 (50.0)	0/16 (0.0)	6/16 (37.5)	0/16 (0.0)	ND	ND
**Abl^4^ clones**	17/25 (68.0)	2/25 (8.0)	15/21 (71.4)	7/21 (33.3)	19/31 (61.3)	15/31 (48.4)
**Wild type clones expressing β-Cat RNAi** c	6/19 (31.6)	0/19 (0.0)	3/24 (12.5)	0/24 (0.0)	ND	ND
**Abl^4^ clones expressing β-Cat RNAi** d	5/8 (62.5)	0/8 (0.0)	6/19 (31.6)	0/19 (0.0)** ^, ^ e	2/15 (13.3)** ^, ^ e	1/15 (6.7)** ^, ^ e

a Testes with CySC clones = testes with GFP^+^, Zfh-1^+^ cells/total testes scored (percentage).

b Testes with >50% marked CySCs = testis where GFP^+^, Zfh-1^+^ cells>GFP^−^, Zfh-1^+^ cells/total testes scores (percentage).

c Wild type clones expressing β-Cat RNAi = Wild type Frt 40A MARCM flies driving β-Cat RNAi specifically in induced clones.

d Abl^4^ clones expressing β-Cat RNAi = Abl^4^ MARCM flies driving β-Cat RNAi specifically in induced clones.

e P value vs Abl^4^ clones.

ND = Not Determined, ACI = After Clone Induction,

* = P value<.05,

** = P value<.01.

Abl has been shown to destabilize β-catenin by phosphorylating a single tyrosine residue (Y667). We hypothesize that loss of Robo2 in CySCs leads to increased Abl activity and increased phosphorylation of β-cateninY667, leading to a destabilization of adherens junctions. Mutations in β-catenin have been generated in which Y667 has been rendered unphosphorylatable (Y667F) or phosphomimetic (Y667E). To determine if the competitiveness of CySCs was affected by the phosphorylation state of β-catenin, we co-expressed wild type β-catenin, unphosphorylatable β-cateninY667F or phosphomimetic β- cateninY667E along with Robo2 RNAi in CySC clones (Table 6). We found that while clones expressing wild type β-catenin and unphosphorylatable β-cateninY667F were maintained at least 8 days ACI, clones expressing phosphomimetic β-cateninY667E were completely lost by 6 days ACI. This indicates that the maintenance of CySCs at the hub is dependent not only on the presence of β-catenin but also the phosphorylation state of β-catenin at Y667.

**Table 6:** The phosphorylation state of β-catenin affects CySC maintenance in the *Drosophila* testis.

Genotype	2 days ACI	6 days ACI	8 days ACI
	**Testes with CySC Clones** a		
**Robo2 RNAi with β- catenin overexpression**	10/33 (30.0)	9/25 (36.0)	10/25 (40.0)
**Robo2 RNAi with β- catenin Y667F overexpression**	12/38 (31.6)	7/27 (25.9)	4/12 (41.6)
**Robo2 RNAi with β- catenin Y667E overexpression**	9/29 (31.0)	0/27 (0.0)*** ^,^ b	0/19 (0.0)** ^,^ b

a Testes with CySC clones = testes with GFP^+^, Zfh-1^+^ cells/total testes scored (percentage).

b P value vs Robo2 RNAi with Arm overexpression.

** = P value<.01.

*** = P value<.001.

### Depletion of Abl in CySCs rescues the Robo2 null phenotype

Since Robo2 and Abl have opposing phenotypes in CySC clones, and both phenotypes can be rescued by modulation of ECad-mediated CySC adhesion to the hub, we considered it likely that Robo2 negatively regulates Abl in CySCs. While Robo and Abl are known to physically interact in the *Drosophila* CNS [29], only a genetic interaction has been shown for Robo2 and Abl [43]. To determine whether Abl and Robo2 genetically interact in the *Drosophila* testis, we asked if simultaneous changes in Robo2 and Abl signaling could balance their phenotypes. Since our data indicate that Robo2 promotes CySC adhesion while Abl opposes it, we reduced the levels of Abl in CySCs lacking *robo2*. At 2 days ACI, we found that while *robo2* null CySCs alone were rarely observed, *robo2* null CySCs were significantly rescued by co-expressing Abl RNAi (Figure 4E, 4F, Table 2). *robo2* null CySCs co-expressing Abl RNAi were often present alongside clonally marked cyst cell daughters (Figure 4F) and remained at 6 days ACI, indicating that rescued CySCs were capable of dividing to produce progeny and that rescued CySCs are maintained over time. We conclude that *robo2* mutant CySCs can be rescued by decreasing *Abl* levels. This further suggests that these two factors have opposing functions in the testis. While we cannot rule out the possibility that Robo2 and Abl may work in parallel pathways, there is prior evidence for this type of genetic interaction between Abl and Robo2 [43]. We propose that Robo2 may attenuate Abl activity in CySCs in order to fine-tune the adhesion levels of CySCs to the niche. Since the Robo2 cytoplasmic domain is not capable of physically interacting with Abl [29], this attenuation likely happens through an indirect mechanism.

The mechanism through which Robo2 and Abl signaling interact is unknown. Using RNA-seq data, we identified several candidate Abl interactors expressed in the *Drosophila* testis [31], [44], including Enabled/Vasp (Ena), Robo, Trio (a Rho GEF), Failed axon connections (Fax) [47], [48], and also members of the Netrin-Frazzled signaling cascade (Table S3). We then used immunofluorescence to confirm niche expression of Robo, Ena and a Fax-GFP fusion protein (Figure S7). To determine if the Robo2 homolog Robo also functions in the testis, we generated mosaic clones of two Robo alleles (*Robo^1^* and *Robo^2^*) which have been previously characterized as genetic nulls [55]. Robo-null CySC clones are lost too rapidly to be detected at 2 days ACI, showing that like Robo2, Robo is required for cyst cell maintenance (Table S4). Future studies will be required to determine the role of Robo and related pathways in the crosstalk between Robo2 and Abl.

### JAK-STAT signaling induces robo2 expression

**Figure 5:**
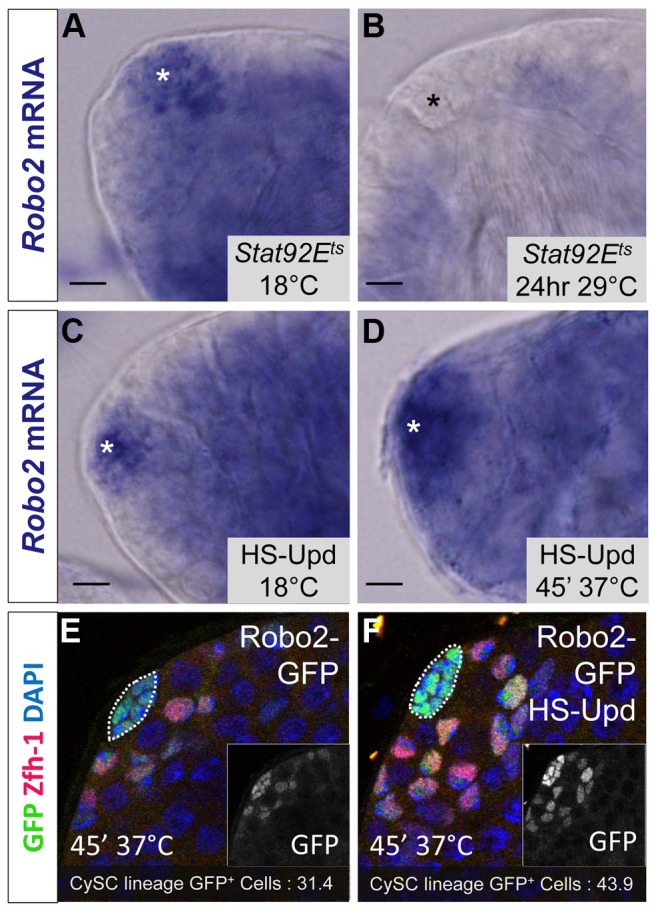
*Robo2* transcription levels in the testis depend on JAK-STAT signaling. (A–D) Whole testis in situ hybridization of *robo2* mRNA. Hubs are marked with an asterisk. In (A) temperature sensitive Stat92E flies (*Stat92E^ts^*) and (C) *hs-Upd* flies at 18°C, *robo2* is expressed in the hub and surrounding early CySCs and daughters, consistent with the wild type *robo2* mRNA expression pattern (not shown). (B) In *Stat92E^ts^* flies shifted to the restrictive temperature (29°C) for 24 hours, *robo2* expression is abolished. (D) In *hs-Upd* flies heat shocked for 45 minutes at 37°C, *robo2* expression expands throughout the testis apex. (E–F) Confocal sections of testes containing the *Robo2-GFP* enhancer trap with Zfh-1 staining CySCs and early cyst cell daughters (red) and GFP reporting *robo2* expression (green). Fewer GFP^+^ cyst lineage cells are found in (E) *Robo2-GFP* testes than in (F) *Robo2-GFP* testes overexpressing Upd. Hubs outlined in white, DAPI stains DNA (blue), scale bars = 10 µm.

While Abl likely modulates adhesion downstream of Slit-Robo signaling in CySCs, little is known in any tissues about the upstream signals controlling the Slit-Robo pathway. Since JAK-STAT signaling is a major regulator of CySC maintenance, is required for ECad-mediated GSC- hub adhesion [15] and has a restricted signaling area similar to the expression pattern of *robo2*, we hypothesized that *robo2* is a transcriptional target of JAK-STAT signaling. To test this idea, we examined *robo2* mRNA levels in testes with altered JAK-STAT signaling. We used established approaches to increase or decrease JAK-STAT signaling and observe subsequent gene expression changes before the induced signaling changes alter normal tissue morphology in the testis niche [18]. Upon temporary (24 hour) downregulation of JAK-STAT signaling in the testis using a temperature sensitive allele of *Stat92E*, we found that *robo2* mRNA expression was not detected by *in situ* staining, while it was clearly detected in flies with unperturbed *Stat92E* expression (Figure 5A, 5B). Conversely, upon upregulation of JAK-STAT signaling through the overexpression of the JAK-STAT ligand Upd, we found that *robo2* mRNA expression expanded in the niche compared to testes with normal levels of JAK-STAT expression (Figure 5C, 5D). It is important to note that while long-term *Upd* overexpression is known to cause an increase in CySC and GSC number in the testis, in this experiment the testes were isolated within 2 hours of initial *Upd* induction, an insufficient time span to allow additional CySCs to accumulate; however we do expect to see changes in gene expression in this short window of *Upd* induction [18]. Because our *in situ* staining was difficult to quantify, we overexpressed Upd using the same technique described above but followed the expression of a *Robo2-GFP* enhancer trap (as shown in Figure 1B). While this enhancer trap may not recapitulate all of the endogenous *robo2* expression pattern, it clearly labels the hub and early somatic cells of the testis niche, allowing us to quantify the number of cells expressing GFP. Following Upd overexpression, we see an expansion of *robo2*-*GFP* expression. Quantification of *robo2-GFP* expressing cells in the CySC lineage [31] revealed a significant increase in the number of *Robo2-GFP*-positive CySCs and early cyst cells following Upd overexpression (43.9 vs. 31.4 cells per testis, p<.0001, n>17), as well as a 38% increase in normalized GFP pixel intensity (p<.01, Figure 5E, 5F). These results further indicate that increased JAK-STAT signaling expands *robo2* expression in the testis. *robo2* may be directly or indirectly activated by Stat92E; distinguishing between these possibilities will require further studies.

Since Stat92E promotes *robo2* expression, and both genes are required for CySC maintenance (Figure 2) [14], [18], [37] we considered that ectopically expressing Robo2 in CySCs lacking *Stat92E* may be sufficient to rescue their loss. However, since Stat92E has many targets in CySCs including *Chinmo* and *Zfh-1*, which are required to maintain CySC fate [3], we expected only a partial rescue of *Stat92E* null CySCs. Consistent with this hypothesis, by comparing *Stat92E* null clones to *Stat92E* null clones overexpressing Robo2, we found that *Stat92E* null clones were significantly rescued by Robo2 expression at 48 hours ACI (Table S5). However, by 60 hours ACI, there was no longer a significant difference in the number of *Stat92E* clones per testis compared to *Stat92E* clones expressing Robo2 per testis (Table S5). This suggests that although re-supplying Robo2 to *Stat92E* null clones slows their loss from the niche, ultimately these clones are not maintained due to the depletion of other Stat92E-dependent factors. We conclude that while Robo2 overexpression allows *Stat92E* null clones to adhere to niche, the clones ultimately fail to self-renew and, as a result, still exit the niche.

## Discussion

This work reveals a novel pathway controlling stem cell competition through stem cell-niche adhesion in the *Drosophila* testis niche. CySCs in the testis niche are constantly competing to ensure that stem cells with improper signaling or adhesion do not remain in the niche. This work highlights the importance of carefully balancing the adhesion levels in stem cells so that the niche can function optimally. We show that the Slit-Robo signaling pathway is required for CySC maintenance in the *Drosophila* testis niche, ensuring that CySCs efficiently adhere to the niche, and that Abl kinase, a putative downstream Slit-Robo signaling component, modulates adhesion levels in CySCs. We also show that *robo2* expression is downstream of JAK-STAT signaling, a major signaling pathway required for the self-renewal of CySCs, thereby establishing a connection between these two stem cell maintenance pathways in the testis.

### The role of Slit-Robo signaling in CySC-niche adhesion

In our model, Slit- Robo2 signaling indirectly attenuates the activity of Abl kinase in CySCs to promote balanced adhesion levels between stem cells and their niche (Figure S8). The well-studied CySC maintenance pathway JAK-STAT controls the expression of *robo2* in CySCs. Interestingly, while the JAK-STAT pathway is required for self-renewal of CySCs and knocking down JAK-STAT signaling in all CySCs leads to a complete loss of this stem cell population, Slit-Robo2 signaling seems to be a primary regulator of stem cell competition. If *robo2* is knocked out of only a few CySCs in the testis niche, the mutant CySCs are rapidly expelled from the niche. However, knockdown of *robo2* in all CySCs does not lead to complete and rapid CySC loss. The presence of wild type CySCs is required to force out less fit *robo2* null cells. This is also the case with ECad; while knockdown of ECad in all CySCs does not result in rapid loss of CySCs from the niche, CySCs with reduced ECad are lost when they must compete for niche space with wild type CySCs. On the other hand, CySCs lacking Abl kinase are overly competitive, forcing wild type CySCs from the niche. This illustrates that stem cells in a single population (for example, CySC) are constantly competing against one another for space in a niche. In our model, adhesion levels can be modulated at multiple regulatory points, and changes in levels of JAK-STAT, Robo2 and Abl activity all lead to alterations in adhesion levels without causing drastic fluctuations in stem cell numbers or function. While *in vivo* fluctuations in Slit-Robo signaling have not been directly shown, both JAK-STAT and ECad levels are known to decline in response to aging [56], leading us to speculate that Slit-Robo may respond to changing niche signals to affect adhesion levels. Because Abl has been shown to directly phosphorylate Robo receptors, including Robo2, leading to attenuation of their activity [29], [57], Abl may be able to feed back on Robo2 to add an additional level of regulation to this signaling network. Understanding the mechanisms underlying stem cell adhesion is particularly important in regenerative medicine, where donor stem cells must efficiently adhere to a host niche in order to engraft [58].

Although the Slit-Robo signaling pathway has been studied primarily in the context of axon guidance, our work, in conjunction with studies in the mammalian hematopoietic stem cell (HSC) niche [30] and the mammalian intestinal stem cell niche [59], establishes the Slit-Robo pathway as a conserved regulator of adult stem cell-niche cell adhesion and competition. In the bone marrow, multiple Robo receptors are expressed in HSCs while Slit ligands are expressed in the endosteal niche, which is comprised of osteoblasts that maintain a long-term population of HSCs [60]–[62]. While the Robo4 receptor is thought to mediate proper HSC adhesion to the niche [30], [61], there is some discrepancy regarding the exact role that Robo4 plays in HSCs [63]. N-cadherin is also required for HSC adhesion to the niche and long term HSC maintenance [64], but its regulation is not understood. Our work suggests that adherens junctions may act downstream of Slit-Robo signaling in HSCs. In addition, since Slit-Robo signaling has roles in gonad development in flies and in the mammalian ovary [65], and high throughput expression studies suggest that this signaling pathway is active in the mammalian testis, [66]–[68] it may function there as well. Data we present in the *Drosophila* testis represent the first mechanistic example of Slit-Robo signaling functioning in an intact adult stem cell niche.

The mechanism we present here may also relate to cancer cells. Alterations in Slit-Robo and Abl kinase activity occur in many cancers and can lead to alterations in adhesion. Changes in Slit activity alter the stability of β-catenin and ECad in both lung and breast cancer cells [69]. In lung cancers, Slit-2 attenuation decreases cell adhesion leading to increased cell migration and metastasis. However in breast cancer cells, overexpression of Slit increases cell adhesion levels, and decreases cell migration [70]. Abl kinase is upregulated in multiple types of leukemia and can lead to decreased cadherin function [71]. Given that Abl misregulation is of great interest with regard to cancer stem cells and their niches, additional studies of Abl function in stem cell competition will be informative.

### The role of Abl kinase adhesion-mediated stem cell competition

We show here that the Abl kinase, in conjunction with Slit-Robo signaling, mediates stem cell competition. Our model suggests that Abl kinase levels can be modulated in individual cells to keep them from losing adhesion to the niche or from becoming overly competitive. This mechanism of regulating adhesion allows for differential regulation of adhesion levels in CySCs and GSCs. Abl mediated CySC competition could also serve as a quality control mechanism in stem cells, since Abl activity is known to be upregulated upon DNA damage [72]. Cells with damaged DNA could have upregulated Abl activity and be outcompeted from the niche due decreased adhesion. These will be interesting areas for future work in the *Drosophila* testis and other stem cell niches.

### Slit-Robo signaling and Abl kinase may play additional adhesion-independent roles in the testis through axon guidance pathways

Not only is the receptor Robo2 required in the testis apex - many additional components of the Robo-Slit pathway are expressed there as well. Furthermore, several Abl interactors, many of which mediate cell migration, are also expressed in the testis. Together these observations suggest that this signaling network may mediate cellular activities in addition to adhesion in the testis niche. Both GSCs and CySCs are dynamic cell populations as revealed by live imaging studies and can form actin-based protrusions and extensions commonly seen in migrating cells [73], [74]. However, characterizing cell migration in intact niches is technically difficult and remains an active area of study. In *Drosophila* male GSCs, a previously characterized Abl interactor, Leukocyte-antigen-related-like (Lar) is required for the regulation of ECad-mediated adhesion although no connection to Abl has been made [75]. Additionally, the profilin gene *chickadee*, which is known to interact with Abl in the *Drosophila* CNS [76], has been implicated in male GSC maintenance through ECad-mediated adhesion of GSCs to the hub as well as encystment of developing spermatogonia by cyst cells [77]. Signaling molecules that belong to other axon guidance signaling cascades in addition to the Slit-Robo pathway are also enriched in the testis apex (Table S3) [44]. Robo signaling has also been implicated in the development of the *Drosophila* gonad [39]. In a normal gonad, somatic gonadal precursors (SGPs), which give rise to CySCs and cyst cells, come together with germline cells and begin to ensheath them. The SGPs and germ cells then come together to form a tight, spherical gonad. Gonads mutant for Slit, Robo and Robo2 display defects in the ensheathment of germ cells by SGPs and gonads fail to undergo proper compaction, resulting in bulbous, misshapen gonads. It will be interesting to determine if any of these axon guidance molecules participate in signaling networks in stem cell niche development and maintenance. Of particular interest is the Netrin-Frazzled pathway, which opposes the activity of the Slit-Robo pathway in the *Drosophila* and mouse CNS midline [78], [79]

### The regulation of Slit-Robo by JAK-STAT in the *Drosophila* testis

The novel connection we observe between the JAK-STAT and Slit-Robo pathways in the *Drosophila* testis is significant since the transcriptional regulation of Slit-Robo signaling components is poorly understood. Our work now links these two highly conserved signaling pathways. Interestingly, within the testis the Slit ligand displays a hub-restricted expression pattern common to only a few signaling ligands including the JAK-STAT and Hedgehog pathway ligands [11], [32]. In this tissue, expression of the Robo2 receptor is also restricted, likely due to its transcriptional control by Stat92E. Restricting both the receptor and ligand to the cells within the testis apex could allow for more precise regulation of signaling in response to small environmental changes. Furthermore, restricted Slit localization may be important for the activity of Robo2 in the testis. In the CNS, Slit is restricted to the midline and repels Robo-expressing axons [25]. While ectopic expression of Slit in the testis does lead to aggregation of CySCs (Figure S1D), this phenotype is difficult to interpret in the absence of loss-of-function data regarding the role of Slit in the testis. Technically, we have not had success in knocking down Slit using RNAi and clonal analysis is not possible in quiescent (hub) cells. Determining the roles of Slit in the niche will be of interest in future work.

While interactions between JAK-STAT and Slit-Robo signaling pathways have not been previously shown, Stat92E is expressed in many of the same tissues as Robo2 during *Drosophila* development including the CNS, cardiac mesoderm and trachea [80]. Additionally, both *robo* and *robo2* transcripts are downregulated in Stat92E null early embryos [81], suggesting that *robo2* may be a target of JAK-STAT in tissues beyond the testis. Furthermore, since both signaling pathway are highly conserved, it will be interesting to determine whether JAK-STAT regulates Slit-Robo signaling in vertebrates. This work sheds light on how stem cell-niche cell adhesion is regulated in an intact niche through the interaction between these highly conserved signaling pathways, and suggests that stem cell niches in other tissues, such as the bone marrow, employ similar mechanisms.

## Materials and Methods

### Fly stocks and culture

Flies were raised on standard yeast/molasses medium at 25°C unless otherwise stated. The following stocks were used: *y w* (wild type), *w*; *lea ^CB02898^/CyO* (A. Spradling), *w; Abl∶GFP* (M. Peifer), *hs-upd* (D. Harrison), *Stat92E^Frankenstein^/Tm3, Sb* and *Stat92E^06346^*/*Tm3,Sb* (both from M. Van Doren), *w; robo2^1^, Frt40a/SM6B* and *w; robo2^8^, Frt40a/SM6B* (*robo2* alleles from D. Van Vactor), *hs-Flp;+; UAS-Shg* (B. Edgar), *hs-FLP, tub-Gal4, UAS –GFP.nls/FM7; neoFRT40A, tub-Gal80* (F. Schweisguth), *hs-FLP, tub-Gal4, UAS-CD8-GFP;+; tub-Gal80, FRT2A* (G. Struhl), *hs-FLP; Frt40A* (A. Spradling), *w; Ubi-GFP.nls, FRT40A; MKRS, hs-FLP/TM6B, Tb*, from Bloomington: *w;+; Frt2A (1997), w;+; Abl^1^, FRT2A/TM6B* (3553), *w;+; Abl^4^, FRT2A/TM6B* (3554), *w; UAS-Abl; Tm2/Tm6B* (8567), *w; UAS-Abl^KinaseDead^; Abl^1^/Tm6B* (8566), *UAS-AblRNAi* ^GL0234^ (35327), from VDRC: *w; UAS-ArmRNAi* (102545) and w; *UAS-ECad RNAi* (8024 and 27081).

### Immunostaining

Testes were dissected, fixed and immunostained as previously described [82] with some exceptions. For Slit staining, Tween20 was substituted for Triton X in washes and block during antibody staining steps, and primary staining was extended to 48 hours. Antibodies used were rabbit anti-Vasa (d-260) (Santa Cruz; 1∶200), rabbit anti-GFP (Torrey Pines; 1∶10,000), rabbit anti-robo2 (B. Dickson; 1∶1000), mouse anti-Slit and mouse anti-Eya (both DSHB; 1∶10), guinea pig anti-Traffic jam (D. Godt; 1∶10,000), and guinea pig anti-Zfh-1 (J. Skeath; 1∶4000). Secondary antibodies were from Molecular Probes and were used at 1∶400 for Alexa 488 conjugated antibodies and 1∶200 for Alexa 568 conjugated antibodies. DNA was counterstained with DAPI (Sigma; 1 µg/ml). Confocal images were acquired with a Zeiss LSM 5 Pascal microscope and figures were assembled with Adobe Photoshop CS5 and Adobe Illustrator CS5.

### Mosaic analysis

The FLP-mediated mitotic recombination technique [83] was used to generate negatively marked homozygous mutant GSC and/or CySC clones for *robo2*. Genotypes used to generate clones are listed in Table S6.

The Mosaic analysis with a repressible cell marker (MARCM) system was used to create positively marked clones for *robo2*, *Ncad* and *Abl* [84]. Genotypes used to generate clones are listed in Table S6. The MARCM system was used for all rescue experiments. Genotypes used for *Robo2*, *Abl*, and *Stat92E* rescue experiments are listed in Table S6. For a mitotic mosaic analysis, adult males were given three 37°C heat shocks of 25 minutes with 25 minutes at 25°C between heat shocks. Following heat shock, flies were kept at 25°C except for rescue experiments when flies were kept at 29°C after heat shock for efficient induction of UAS constructs. For clones expressing βcatenin phosphorylation variants, adult males were given three 37°C heat shocks of 30 minutes with 30 minutes at 25°C between heat shocks Flies were dissected and stained at specified days ACI. All controls for mosaic analysis were age-matched for each experiment and done in parallel.

### In situ hybridization

Probes for in situ hybridization were generated from *robo2* cDNA obtained from *Drosophila* Genomics Resource Center (clone RE21729) and linearized with NotI. Digoxigenin-labeled anti-sense RNA probe was transcribed in vitro using T3 RNA polymerase according to the manufacturer's instructions (Roche). In situ hybridizations were performed as described [85] and visualized with an Olympus BX51 microscope.

### Inducing or removing JAK-STAT signaling

Ectopic JAK-STAT signaling was induced via *Upd* overexpression by a 45 minute heat shock at 37°C of male flies carrying *hs-Upd* (D. Harrison) or *hs-Upd/Lea^CB02898^* with *Lea^CB02898^/CyO* males as heat shock controls. JAK-STAT signaling was abolished by shifting *stat92E* temperature-sensitive flies (*stat92EFrankenstein/stat92E06346*) from the permissive temperature (18°C) to the restrictive temperature (29°C) for 1 day.

Additional details regarding methods can be found in Protocol S1.

## Supporting Information

Figure S1 Robo2 or Slit overexpression is not sufficient for ectopic stem cell self-renewal. (A–B) Confocal sections of adult testes with vasa staining the germline lineage (red), traffic jam staining the hub and cyst lineage (green) and DAPI staining DNA (blue). (A) In Gal4 driver controls (C587-Gal4), GSCs (arrowhead) and CySCs (white arrow) are brightly stained with vasa and traffic jam respectively. As cells differentiate, vasa and traffic jam staining becomes dimmer and spermatocytes with larger, less compact nuclei are present (yellow arrow). (B) When Robo2 is overexpressed in the CySC lineage for 7 days via the C587-Gal4 driver, testes look similar to driver controls. GSCs (arrowhead) and CySCs (white arrow) are present by the hub and differentiating spermatocytes can be identified away from the hub. No stem cell overproliferation is obvious outside of the niche. (C–D) Confocal sections of adult testes with vasa staining the germline lineage (red), Zfh-1 staining CySCs and early cyst cell daughters (green) and DAPI staining DNA. (C) In Gal4 driver controls (Ubi-Gal4), testes contain GSCs with bright vasa staining in contact with the hub dispersed with Zfh-1 positive CySCs (white arrows). Testes look similar to (A). In (D), testes ubiquitously overexpressing Slit via the Ubi-Gal4 driver have aggregates of Zfh-1 positive cells (dashed yellow outlines) in contact with the hub, and Zfh-1 cells are clumped together rather than dispersed throughout the germline. Inset, lower confocal plane of testis in D, just below the hub. Hubs outlined in white. Scale bars = 10 µm.
(TIF)

Figure S2 *Robo2* is not required for stem cell viability in the *Drosophila* testis niche. (A–B) Confocal sections of adult testes with TUNEL labeling apoptotic cells (Red) and DNA stained with DAPI (blue). Testes contain high levels of (A) wild type control clones or (B) *robo2^1^* mutant clones. Dying single cells are rarely detected within two cell nuclei or approximately 10 microns from the hub (area denoted with dashed yellow circle) in either genotype. Both genotypes contain apoptotic spermatogonial cysts (arrows), as expected. Hubs outlined in white. Scale bars = 10 µm.
(TIF)

Figure S3 Knockdown of Robo2 in the entire CySC population does not lead to rapid CySC loss. (A–B) Confocal section of an adult testis with vasa staining the germline lineage (red), Armadillo and 1B1 staining hub cells and fusome respectively (green) and DAPI staining DNA (blue). (A) When Robo2 RNAi is expressed in CySCs and cyst cells of adult flies (0–5 days post eclosion) for 7 days using the C587-Gal4 driver in conjunction with Gal80^TS^, both GSCs (arrowheads) and CySCs (arrows) remain in the niche. Differentiating cyst cells and spermatogonia are evident away from the hub. (B) When Robo2 RNAi is expressed in the CySC lineage during development and adulthood using the C587-Gal4 driver, GSCs are lost from the niche (single GSC, arrowhead) and CySCs are present but not associated with GSCs (arrows). Differentiating spermatogonia appear grossly abnormal.
(TIF)

Figure S4 Adhesion complex factors are expressed in the testis and enriched in the niche. (A–C) Confocal sections of adult testes stained with DAPI (blue) and (A) anti-N-cadherin (green), (B) anti-E-cadherin (green) or (C) anti- β-catenin (green). N-cadherin, E-cadherin and β-catenin have similar hub enriched expression patterns. (A′, B′ and C′ show green channel alone.)
(TIF)

Figure S5 Knockdown of ECad in the entire CySC population does not lead to rapid CySC loss. Confocal section of an adult testis with traffic jam staining the hub and cyst lineage (red), Armadillo and 1B1 staining hub cells and fusome respectively (green) and DAPI staining DNA (blue). When ECad RNAi is expressed in the CySC lineage for 7 days via the C587-Gal4 driver, both GSCs (arrowheads) and CySCs (arrows) remain in the niche and testes appear grossly normal. Differentiating cyst cells and spermatogonia (identified by elongated fusomes) are evident away from the hub.
(TIF)

Figure S6 Clonal overexpression of Abl RNAi or ECad is sufficient to cause CySCs to outcompete their neighbors. (A–C) Confocal sections of testes with Zfh-1 staining CySCs and early cyst cell daughters (red). Positively marked mosaic clones are identified by presence of GFP (green). At 8 days ACI, (A) wild type mosaic testes contain a small number of marked CySCs per testis (arrowhead). Testes containing clones overexpressing (B) Abl RNAi or (C) ECad. Marked CySC outcompete their neighbors and testes contain many marked CySCs (arrowheads). Insets, GFP channel alone.
(TIF)

Figure S7 Factors involved in Abl kinase signaling are expressed in the testis apex. (A) Confocal section of an adult testis immunostained with DAPI (blue) and (A) anti-Robo (green) or (B) anti-Enabled (green). (A) Robo is expressed at hub-stem cell contacts. (A′) Robo staining alone (B) Enabled is expressed around hub cells and at hub-stem cell contacts. (B′) Enabled staining alone. (C) Confocal section of an adult testis expressing a Fax-GFP protein fusion stained with anti-GFP (green) and DAPI (blue). Fax is enriched in the hub and CySC lineage. (C′) GFP staining alone. Scale bars = 10 µm.
(TIF)

Figure S8 Model of JAK-STAT and Slit-Robo signaling interacting to control adhesion levels in the *Drosophila* testis niche. The axon guidance receptor Robo2 (orange) is required to regulate the level of adhesion in CySCs. The Robo2 ligand Slit (red) is expressed in the hub. Robo2 genetically interacts with Abl (green), antagonizing Abl activity to modulate adhesion levels. Abl can phosphorylate β-cat (light green), leading to the destabilization of the β-cat/ECad adherens junction complex and a decrease in cell adhesion levels. *Robo2* transcription is controlled by the transcription factor Stat92E (yellow). Stat92E is activated by the Upd ligand (light purple), which is secreted from the hub and activates JAK-STAT signaling. Signaling in GSCs is not shown.
(TIF)

Table S1 Robo2 is not required cell-autonomously for GSC maintenance in the *Drosophila* testis.
(DOCX)

Table S2 Ncad is not required to maintain CySC clones in the *Drosophila* testis.
(DOCX)

Table S3 Members of the Slit-Robo signaling pathway are expressed in stem cell enriched testes.
(DOCX)

Table S4 CySCs lacking Robo are rapidly lost from the *Drosophila* testis niche.
(DOCX)

Table S5 *Stat92E* null clones are temporarily rescued by overexpression of Robo2.
(DOCX)

Table S6 Full genotypes for mosaic analysis.
(DOCX)

Protocol S1 Detailed methods, antibody and fly stock information.
(DOCX)

## References

[1] Lander AD, Kimble J, Clevers H, Fuchs E, Montarras D, et al (2012) What does the concept of the stem cell niche really mean today? BMC Biol 10: 19. 22405133 10.1186/1741-7007-10-19 PMC3298504

[2] Chen S, Lewallen M, Xie T (2013) Adhesion in the stem cell niche: biological roles and regulation. Development 140: 255–265. 23250203 10.1242/dev.083139 PMC3597204

[3] Matunis EL, Stine RR, de Cuevas M (2012) Recent advances in Drosophila male germline stem cell biology. Spermatogenesis 2: 137–144. 23087833 10.4161/spmg.21763 PMC3469437

[4] Voog J, D'Alterio C, Jones DL (2008) Multipotent somatic stem cells contribute to the stem cell niche in the Drosophila testis. Nature 454: 1132–1136. 18641633 10.1038/nature07173 PMC2599791

[5] Yamashita YM, Jones DL, Fuller MT (2003) Orientation of asymmetric stem cell division by the APC tumor suppressor and centrosome. Science 301: 1547–1550. 12970569 10.1126/science.1087795

[6] Sheng XR, Matunis E (2011) Live imaging of the Drosophila spermatogonial stem cell niche reveals novel mechanisms regulating germline stem cell output. Development 138: 3367–3376. 21752931 10.1242/dev.065797 PMC3143561

[7] Hardy RW, Tokuyasu KT, Lindsley DL, Garavito M (1979) The germinal proliferation center in the testis of Drosophila melanogaster. J Ultrastruct Res 69: 180–190. 114676 10.1016/s0022-5320(79)90108-4

[8] Kiger AA, White-Cooper H, Fuller MT (2000) Somatic support cells restrict germline stem cell self-renewal and promote differentiation. Nature 407: 750–754. 11048722 10.1038/35037606

[9] Tran J, Brenner TJ, DiNardo S (2000) Somatic control over the germline stem cell lineage during Drosophila spermatogenesis. Nature 407: 754–757. 11048723 10.1038/35037613

[10] Schulz C, Wood CG, Jones DL, Tazuke SI, Fuller MT (2002) Signaling from germ cells mediated by the rhomboid homolog stet organizes encapsulation by somatic support cells. Development 129: 4523–4534. 12223409 10.1242/dev.129.19.4523

[11] Tulina N, Matunis E (2001) Control of stem cell self-renewal in Drosophila spermatogenesis by JAK-STAT signaling. Science (New York, NY) 294: 2546–2549. 10.1126/science.1066700 11752575

[12] Kiger AA, Jones DL, Schulz C, Rogers MB, Fuller MT (2001) Stem cell self-renewal specified by JAK-STAT activation in response to a support cell cue. Science (New York, NY) 294: 2542–2545. 10.1126/science.1066707 11752574

[13] Flaherty MS, Salis P, Evans CJ, Ekas LA, Marouf A, et al (2010) chinmo is a functional effector of the JAK/STAT pathway that regulates eye development, tumor formation, and stem cell self-renewal in Drosophila. Dev Cell 18: 556–568. 20412771 10.1016/j.devcel.2010.02.006 PMC2859208

[14] Leatherman JL, Dinardo S (2008) Zfh-1 controls somatic stem cell self-renewal in the Drosophila testis and nonautonomously influences germline stem cell self-renewal. Cell Stem Cell 3: 44–54. 18593558 10.1016/j.stem.2008.05.001 PMC2601693

[15] Leatherman JL, Dinardo S (2010) Germline self-renewal requires cyst stem cells and stat regulates niche adhesion in Drosophila testes. Nat Cell Biol 12: 806–811. 20622868 10.1038/ncb2086 PMC2917891

[16] Stine RR, Matunis EL (2013) Stem cell competition: finding balance in the niche. Trends Cell Biol 10.1016/j.tcb.2013.03.001 PMC3729747 23597843

[17] Jin Z, Kirilly D, Weng C, Kawase E, Song X, et al (2008) Differentiation-defective stem cells outcompete normal stem cells for niche occupancy in the Drosophila ovary. Cell Stem Cell 2: 39–49. 18371420 10.1016/j.stem.2007.10.021 PMC8387725

[18] Issigonis M, Tulina N, de Cuevas M, Brawley C, Sandler L, et al (2009) JAK-STAT signal inhibition regulates competition in the Drosophila testis stem cell niche. Science 326: 153–156. 19797664 10.1126/science.1176817 PMC3073347

[19] Lehmann R (2012) Germline stem cells: origin and destiny. Cell Stem Cell 10: 729–739. 22704513 10.1016/j.stem.2012.05.016 PMC3750984

[20] Ypsilanti AR, Zagar Y, Chedotal A (2010) Moving away from the midline: new developments for Slit and Robo. Development 137: 1939–1952. 20501589 10.1242/dev.044511

[21] Simpson JH, Bland KS, Fetter RD, Goodman CS (2000) Short-range and long-range guidance by Slit and its Robo receptors: a combinatorial code of Robo receptors controls lateral position. Cell 103: 1019–1032. 11163179 10.1016/s0092-8674(00)00206-3

[22] Simpson JH, Kidd T, Bland KS, Goodman CS (2000) Short-range and long-range guidance by slit and its Robo receptors. Robo and Robo2 play distinct roles in midline guidance. Neuron 28: 753–766. 11163264 10.1016/s0896-6273(00)00151-3

[23] Rajagopalan S, Vivancos V, Nicolas E, Dickson BJ (2000) Selecting a longitudinal pathway: Robo receptors specify the lateral position of axons in the Drosophila CNS. Cell 103: 1033–1045. 11163180 10.1016/s0092-8674(00)00207-5

[24] Rajagopalan S, Nicolas E, Vivancos V, Berger J, Dickson BJ (2000) Crossing the midline: roles and regulation of Robo receptors. Neuron 28: 767–777. 11163265 10.1016/s0896-6273(00)00152-5

[25] Kidd T, Bland KS, Goodman CS (1999) Slit is the midline repellent for the robo receptor in Drosophila. Cell 96: 785–794. 10102267 10.1016/s0092-8674(00)80589-9

[26] Rhee J, Buchan T, Zukerberg L, Lilien J, Balsamo J (2007) Cables links Robo-bound Abl kinase to N-cadherin-bound beta-catenin to mediate Slit-induced modulation of adhesion and transcription. Nat Cell Biol 9: 883–892. 17618275 10.1038/ncb1614

[27] Prasad A, Qamri Z, Wu J, Ganju RK (2007) Slit-2/Robo-1 modulates the CXCL12/CXCR4-induced chemotaxis of T cells. J Leukoc Biol 82: 465–476. 17565045 10.1189/jlb.1106678 PMC2286829

[28] Santiago-Martinez E, Soplop NH, Patel R, Kramer SG (2008) Repulsion by Slit and Roundabout prevents Shotgun/E-cadherin-mediated cell adhesion during Drosophila heart tube lumen formation. J Cell Biol 182: 241–248. 18663139 10.1083/jcb.200804120 PMC2483515

[29] Bashaw GJ, Kidd T, Murray D, Pawson T, Goodman CS (2000) Repulsive axon guidance: Abelson and Enabled play opposing roles downstream of the roundabout receptor. Cell 101: 703–715. 10892742 10.1016/s0092-8674(00)80883-1

[30] Smith-Berdan S, Nguyen A, Hassanein D, Zimmer M, Ugarte F, et al (2011) Robo4 cooperates with CXCR4 to specify hematopoietic stem cell localization to bone marrow niches. Cell Stem Cell 8: 72–83. 21211783 10.1016/j.stem.2010.11.030 PMC3625377

[31] Buszczak M, Paterno S, Lighthouse D, Bachman J, Planck J, et al (2007) The carnegie protein trap library: a versatile tool for Drosophila developmental studies. Genetics 175: 1505–1531. 17194782 10.1534/genetics.106.065961 PMC1840051

[32] Michel M, Kupinski AP, Raabe I, Bokel C (2012) Hh signalling is essential for somatic stem cell maintenance in the Drosophila testis niche. Development 139: 2663–2669. 22745310 10.1242/dev.075242

[33] Spitzweck B, Brankatschk M, Dickson BJ (2010) Distinct protein domains and expression patterns confer divergent axon guidance functions for Drosophila Robo receptors. Cell 140: 409–420. 20144763 10.1016/j.cell.2010.01.002

[34] Lee CH, Herman T, Clandinin TR, Lee R, Zipursky SL (2001) N-cadherin regulates target specificity in the Drosophila visual system. Neuron 30: 437–450. 11395005 10.1016/s0896-6273(01)00291-4

[35] Fabrizio JJ, Boyle M, DiNardo S (2003) A somatic role for eyes absent (eya) and sine oculis (so) in Drosophila spermatocyte development. Dev Biol 258: 117–128. 12781687 10.1016/s0012-1606(03)00127-1

[36] Issigonis M, Matunis E (2012) The Drosophila BCL6 homolog Ken and Barbie promotes somatic stem cell self-renewal in the testis niche. Dev Biol 368: 181–192. 22580161 10.1016/j.ydbio.2012.04.034 PMC3402624

[37] Brawley C, Matunis E (2004) Regeneration of male germline stem cells by spermatogonial dedifferentiation in vivo. Science 304: 1331–1334. 15143218 10.1126/science.1097676

[38] Yacobi-Sharon K, Namdar Y, Arama E (2013) Alternative Germ Cell Death Pathway in Drosophila Involves HtrA2/Omi, Lysosomes, and a Caspase-9 Counterpart. Dev Cell 25: 29–42. 23523076 10.1016/j.devcel.2013.02.002

[39] Weyers JJ, Milutinovich AB, Takeda Y, Jemc JC, Van Doren M (2011) A genetic screen for mutations affecting gonad formation in Drosophila reveals a role for the slit/robo pathway. Dev Biol 353: 217–228. 21377458 10.1016/j.ydbio.2011.02.023 PMC3635084

[40] Gilboa L, Lehmann R (2006) Soma-germline interactions coordinate homeostasis and growth in the Drosophila gonad. Nature 443: 97–100. 16936717 10.1038/nature05068

[41] Le Bras S, Van Doren M (2006) Development of the male germline stem cell niche in Drosophila. Dev Biol 294: 92–103. 16566915 10.1016/j.ydbio.2006.02.030

[42] Wang X, Pan L, Wang S, Zhou J, McDowell W, et al (2011) Histone H3K9 trimethylase Eggless controls germline stem cell maintenance and differentiation. PLoS Genet 7: e1002426. 22216012 10.1371/journal.pgen.1002426 PMC3245301

[43] Wills Z, Emerson M, Rusch J, Bikoff J, Baum B, et al (2002) A Drosophila homolog of cyclase-associated proteins collaborates with the Abl tyrosine kinase to control midline axon pathfinding. Neuron 36: 611–622. 12441051 10.1016/s0896-6273(02)01022-x

[44] Gan Q, Chepelev I, Wei G, Tarayrah L, Cui K, et al (2010) Dynamic regulation of alternative splicing and chromatin structure in Drosophila gonads revealed by RNA-seq. Cell Res 20: 763–783. 20440302 10.1038/cr.2010.64 PMC2919574

[45] Fox DT, Peifer M (2007) Abelson kinase (Abl) and RhoGEF2 regulate actin organization during cell constriction in Drosophila. Development 134: 567–578. 17202187 10.1242/dev.02748

[46] Smith JA, Liebl EC (2005) Identification of the molecular lesions in alleles of the Drosophila Abelson tyrosine kinase. Drosophila Information Service 88: 20–22.

[47] Lanier LM, Gertler FB (2000) From Abl to actin: Abl tyrosine kinase and associated proteins in growth cone motility. Curr Opin Neurobiol 10: 80–87. 10679439 10.1016/s0959-4388(99)00058-6

[48] Hernandez SE, Krishnaswami M, Miller AL, Koleske AJ (2004) How do Abl family kinases regulate cell shape and movement? Trends Cell Biol 14: 36–44. 14729179 10.1016/j.tcb.2003.11.003

[49] Huang J, Kalderon D (2014) Coupling of Hedgehog and Hippo pathways promotes stem cell maintenance by stimulating proliferation. J Cell Biol 205: 325–338. 24798736 10.1083/jcb.201309141 PMC4018789

[50] Amoyel M, Simons BD, Bach EA (2014) Neutral competition of stem cells is skewed by proliferative changes downstream of Hh and Hpo. EMBO J 10.15252/embj.201387500 PMC4253521 25092766

[51] Gambacorti-Passerini C, le Coutre P, Mologni L, Fanelli M, Bertazzoli C, et al (1997) Inhibition of the ABL kinase activity blocks the proliferation of BCR/ABL+ leukemic cells and induces apoptosis. Blood Cells Mol Dis 23: 380–394. 9446752 10.1006/bcmd.1997.0155

[52] Tamada M, Farrell DL, Zallen JA (2012) Abl regulates planar polarized junctional dynamics through beta-catenin tyrosine phosphorylation. Dev Cell 22: 309–319. 22340496 10.1016/j.devcel.2011.12.025 PMC3327890

[53] Singh J, Aaronson SA, Mlodzik M (2010) Drosophila Abelson kinase mediates cell invasion and proliferation through two distinct MAPK pathways. Oncogene 29: 4033–4045. 20453880 10.1038/onc.2010.155 PMC2919309

[54] Horn T, Arziman Z, Berger J, Boutros M (2007) GenomeRNAi: a database for cell-based RNAi phenotypes. Nucleic Acids Res 35: D492–497. 17135194 10.1093/nar/gkl906 PMC1747177

[55] Seeger M, Tear G, Ferres-Marco D, Goodman CS (1993) Mutations affecting growth cone guidance in Drosophila: genes necessary for guidance toward or away from the midline. Neuron 10: 409–426. 8461134 10.1016/0896-6273(93)90330-t

[56] Toledano H, D'Alterio C, Czech B, Levine E, Jones DL (2012) The let-7-Imp axis regulates ageing of the Drosophila testis stem-cell niche. Nature 485: 605–610. 22660319 10.1038/nature11061 PMC4829122

[57] Boyle M, Wong C, Rocha M, Jones DL (2007) Decline in self-renewal factors contributes to aging of the stem cell niche in the Drosophila testis. Cell Stem Cell 1: 470–478. 18371382 10.1016/j.stem.2007.08.002

[58] Sahin AO, Buitenhuis M (2012) Molecular mechanisms underlying adhesion and migration of hematopoietic stem cells. Cell Adh Migr 6: 39–48. 22647939 10.4161/cam.18975 PMC3364136

[59] Zhou WJ, Geng ZH, Spence JR, Geng JG (2013) Induction of intestinal stem cells by R-spondin 1 and Slit2 augments chemoradioprotection. Nature 501: 107–111. 23903657 10.1038/nature12416 PMC3888063

[60] Smith-Berdan S, Schepers K, Ly A, Passegue E, Forsberg EC (2012) Dynamic expression of the Robo ligand Slit2 in bone marrow cell populations. Cell Cycle 11: 675–682. 22313734 10.4161/cc.11.4.19146

[61] Shibata F, Goto-Koshino Y, Morikawa Y, Komori T, Ito M, et al (2009) Roundabout 4 is expressed on hematopoietic stem cells and potentially involved in the niche-mediated regulation of the side population phenotype. Stem Cells 27: 183–190. 18927479 10.1634/stemcells.2008-0292 PMC2883560

[62] Geutskens SB, Andrews WD, van Stalborch AM, Brussen K, Holtrop-de Haan SE, et al (2012) Control of human hematopoietic stem/progenitor cell migration by the extracellular matrix protein Slit3. Lab Invest 92: 1129–1139. 22614124 10.1038/labinvest.2012.81

[63] Goto-Koshino Y, Fukuchi Y, Shibata F, Abe D, Kuroda K, et al (2012) Robo4 plays a role in bone marrow homing and mobilization, but is not essential in the long-term repopulating capacity of hematopoietic stem cells. PLoS One 7: e50849. 23226403 10.1371/journal.pone.0050849 PMC3511340

[64] Hosokawa K, Arai F, Yoshihara H, Iwasaki H, Nakamura Y, et al (2010) Knockdown of N-cadherin suppresses the long-term engraftment of hematopoietic stem cells. Blood 116: 554–563. 20427705 10.1182/blood-2009-05-224857

[65] Dickinson RE, Duncan WC (2010) The SLIT-ROBO pathway: a regulator of cell function with implications for the reproductive system. Reproduction 139: 697–704. 20100881 10.1530/REP-10-0017 PMC2971463

[66] Dalkic E, Kuscu C, Sucularli C, Aydin IT, Akcali KC, et al (2006) Alternatively spliced Robo2 isoforms in zebrafish and rat. Dev Genes Evol 216: 555–563. 16625395 10.1007/s00427-006-0070-y

[67] Rolland AD, Lehmann KP, Johnson KJ, Gaido KW, Koopman P (2011) Uncovering gene regulatory networks during mouse fetal germ cell development. Biol Reprod 84: 790–800. 21148109 10.1095/biolreprod.110.088443 PMC3062041

[68] Nef S, Schaad O, Stallings NR, Cederroth CR, Pitetti JL, et al (2005) Gene expression during sex determination reveals a robust female genetic program at the onset of ovarian development. Dev Biol 287: 361–377. 16214126 10.1016/j.ydbio.2005.09.008

[69] Tseng RC, Lee SH, Hsu HS, Chen BH, Tsai WC, et al (2010) SLIT2 attenuation during lung cancer progression deregulates beta-catenin and E-cadherin and associates with poor prognosis. Cancer Res 70: 543–551. 20068157 10.1158/0008-5472.CAN-09-2084

[70] Prasad A, Paruchuri V, Preet A, Latif F, Ganju RK (2008) Slit-2 induces a tumor-suppressive effect by regulating beta-catenin in breast cancer cells. J Biol Chem 283: 26624–26633. 18611862 10.1074/jbc.M800679200 PMC2546548

[71] O'Leary H, Akers SM, Piktel D, Walton C, Fortney JE, et al (2010) VE-cadherin Regulates Philadelphia Chromosome Positive Acute Lymphoblastic Leukemia Sensitivity to Apoptosis. Cancer Microenviron 3: 67–81. 21209775 10.1007/s12307-010-0035-6 PMC2990486

[72] Maiani E, Diederich M, Gonfloni S (2011) DNA damage response: the emerging role of c-Abl as a regulatory switch? Biochem Pharmacol 82: 1269–1276. 21763684 10.1016/j.bcp.2011.07.001

[73] Sheng XR, Brawley CM, Matunis EL (2009) Dedifferentiating spermatogonia outcompete somatic stem cells for niche occupancy in the Drosophila testis. Cell Stem Cell 5: 191–203. 19664993 10.1016/j.stem.2009.05.024 PMC2750779

[74] Cheng J, Tiyaboonchai A, Yamashita YM, Hunt AJ (2011) Asymmetric division of cyst stem cells in Drosophila testis is ensured by anaphase spindle repositioning. Development 138: 831–837. 21303845 10.1242/dev.057901 PMC3035088

[75] Srinivasan S, Mahowald AP, Fuller MT (2012) The receptor tyrosine phosphatase Lar regulates adhesion between Drosophila male germline stem cells and the niche. Development 139: 1381–1390. 22378638 10.1242/dev.070052 PMC3308176

[76] Wills Z, Marr L, Zinn K, Goodman CS, Van Vactor D (1999) Profilin and the Abl tyrosine kinase are required for motor axon outgrowth in the Drosophila embryo. Neuron 22: 291–299. 10069335 10.1016/s0896-6273(00)81090-9

[77] Shields AR, Spence AC, Yamashita YM, Davies EL, Fuller MT (2014) The actin-binding protein profilin is required for germline stem cell maintenance and germ cell enclosure by somatic cyst cells. Development 141: 73–82. 24346697 10.1242/dev.101931 PMC3865751

[78] Bashaw GJ, Goodman CS (1999) Chimeric axon guidance receptors: the cytoplasmic domains of slit and netrin receptors specify attraction versus repulsion. Cell 97: 917–926. 10399919 10.1016/s0092-8674(00)80803-x

[79] Causeret F, Danne F, Ezan F, Sotelo C, Bloch-Gallego E (2002) Slit antagonizes netrin-1 attractive effects during the migration of inferior olivary neurons. Dev Biol 246: 429–440. 12051827 10.1006/dbio.2002.0681

[80] Li J, Li W, Calhoun HC, Xia F, Gao FB, et al (2003) Patterns and functions of STAT activation during Drosophila embryogenesis. Mech Dev 120: 1455–1468. 14654218 10.1016/j.mod.2003.09.004 PMC3090291

[81] Tsurumi A, Xia F, Li J, Larson K, LaFrance R, et al (2011) STAT is an essential activator of the zygotic genome in the early Drosophila embryo. PLoS Genet 7: e1002086. 21637778 10.1371/journal.pgen.1002086 PMC3102735

[82] Matunis E, Tran J, Gonczy P, Caldwell K, DiNardo S (1997) punt and schnurri regulate a somatically derived signal that restricts proliferation of committed progenitors in the germline. Development 124: 4383–4391. 9334286 10.1242/dev.124.21.4383

[83] Xu T, Rubin GM (1993) Analysis of genetic mosaics in developing and adult Drosophila tissues. Development 117: 1223–1237. 8404527 10.1242/dev.117.4.1223

[84] Lee T, Luo L (1999) Mosaic analysis with a repressible cell marker for studies of gene function in neuronal morphogenesis. Neuron 22: 451–461. 10197526 10.1016/s0896-6273(00)80701-1

[85] Terry NA, Tulina N, Matunis E, DiNardo S (2006) Novel regulators revealed by profiling Drosophila testis stem cells within their niche. Dev Biol 294: 246–257. 16616121 10.1016/j.ydbio.2006.02.048

