# Supplementary figures and images for: Coordinate Regulation of Stem Cell Competition by Slit-Robo and JAK-STAT Signaling in the Drosophila Testis
Source: PLoS Genet. 2014 Nov 6;10(11):e1004713. doi: 10.1371/journal.pgen.1004713 (PMC4222695; doi:10.1371/journal.pgen.1004713)

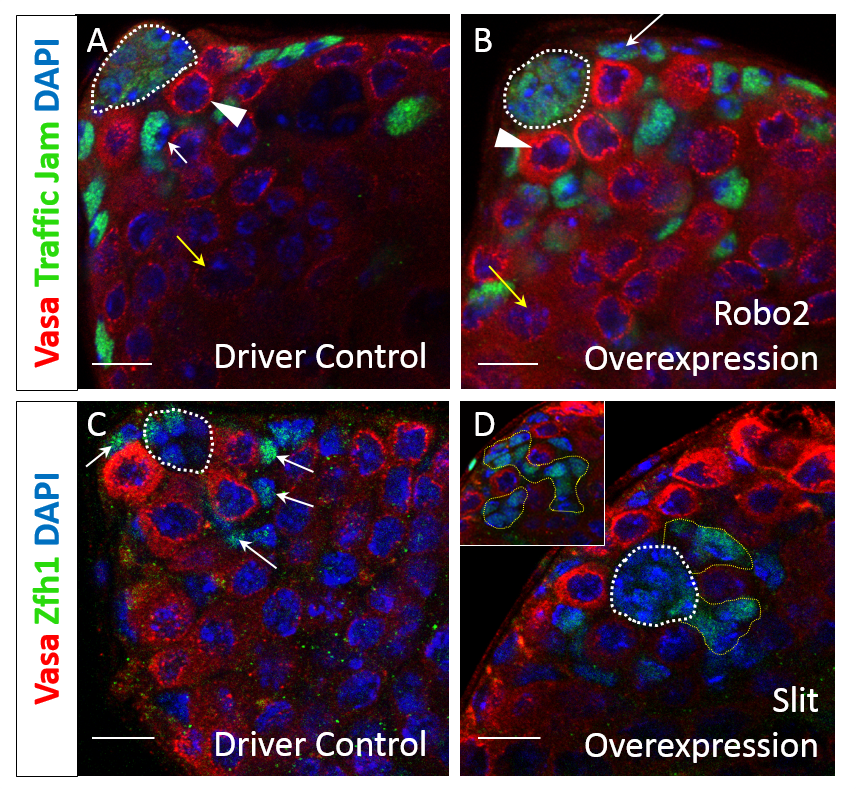

Supplement: Figure S1 — Robo2 or Slit overexpression is not sufficient for ectopic stem cell self-renewal. (A–B) Confocal sections of adult testes with vasa staining the germline lineage (red), traffic jam staining the hub and cyst lineage (green) and DAPI staining DNA (blue). (A) In Gal4 driver controls (C587-Gal4), GSCs (arrowhead) and CySCs (white arrow) are brightly stained with vasa and traffic jam respectively. As cells differentiate, vasa and traffic jam staining becomes dimmer and spermatocytes with larger, less compact nuclei are present (yellow arrow). (B) When Robo2 is overexpressed in the CySC lineage for 7 days via the C587-Gal4 driver, testes look similar to driver controls. GSCs (arrowhead) and CySCs (white arrow) are present by the hub and differentiating spermatocytes can be identified away from the hub. No stem cell overproliferation is obvious outside of the niche. (C–D) Confocal sections of adult testes with vasa staining the germline lineage (red), Zfh-1 staining CySCs and early cyst cell daughters (green) and DAPI staining DNA. (C) In Gal4 driver controls (Ubi-Gal4), testes contain GSCs with bright vasa staining in contact with the hub dispersed with Zfh-1 positive CySCs (white arrows). Testes look similar to (A). In (D), testes ubiquitously overexpressing Slit via the Ubi-Gal4 driver have aggregates of Zfh-1 positive cells (dashed yellow outlines) in contact with the hub, and Zfh-1 cells are clumped together rather than dispersed throughout the germline. Inset, lower confocal plane of testis in D, just below the hub. Hubs outlined in white. Scale bars = 10 µm. (TIF) [file pgen.1004713.s001.tif]

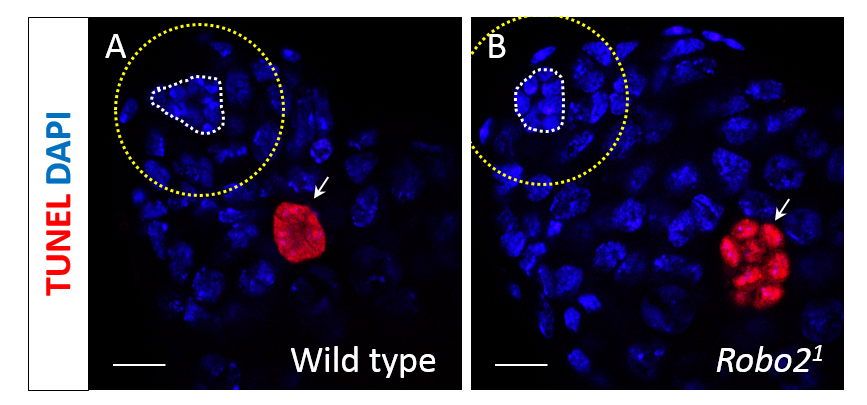

Supplement: Figure S2 — Robo2 is not required for stem cell viability in the Drosophila testis niche. (A–B) Confocal sections of adult testes with TUNEL labeling apoptotic cells (Red) and DNA stained with DAPI (blue). Testes contain high levels of (A) wild type control clones or (B) robo21 mutant clones. Dying single cells are rarely detected within two cell nuclei or approximately 10 microns from the hub (area denoted with dashed yellow circle) in either genotype. Both genotypes contain apoptotic spermatogonial cysts (arrows), as expected. Hubs outlined in white. Scale bars = 10 µm. (TIF) [file pgen.1004713.s002.tif]

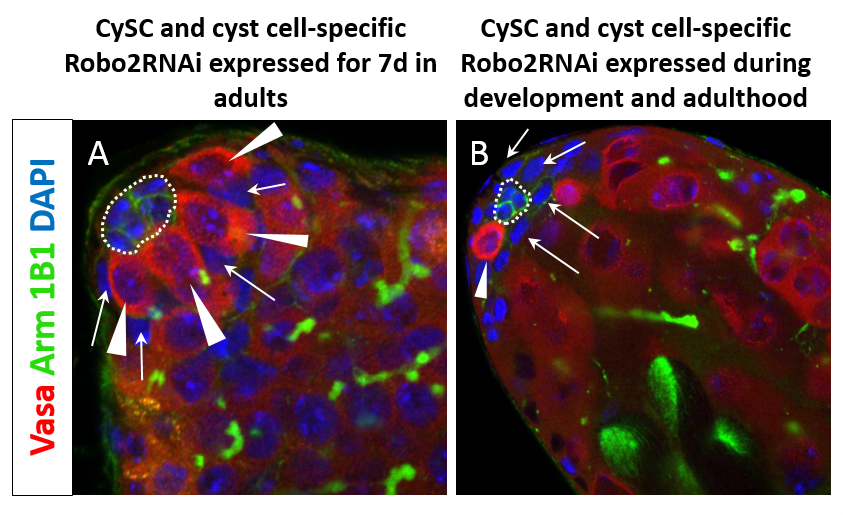

Supplement: Figure S3 — Knockdown of Robo2 in the entire CySC population does not lead to rapid CySC loss. (A–B) Confocal section of an adult testis with vasa staining the germline lineage (red), Armadillo and 1B1 staining hub cells and fusome respectively (green) and DAPI staining DNA (blue). (A) When Robo2 RNAi is expressed in CySCs and cyst cells of adult flies (0–5 days post eclosion) for 7 days using the C587-Gal4 driver in conjunction with Gal80TS, both GSCs (arrowheads) and CySCs (arrows) remain in the niche. Differentiating cyst cells and spermatogonia are evident away from the hub. (B) When Robo2 RNAi is expressed in the CySC lineage during development and adulthood using the C587-Gal4 driver, GSCs are lost from the niche (single GSC, arrowhead) and CySCs are present but not associated with GSCs (arrows). Differentiating spermatogonia appear grossly abnormal. (TIF) [file pgen.1004713.s003.tif]

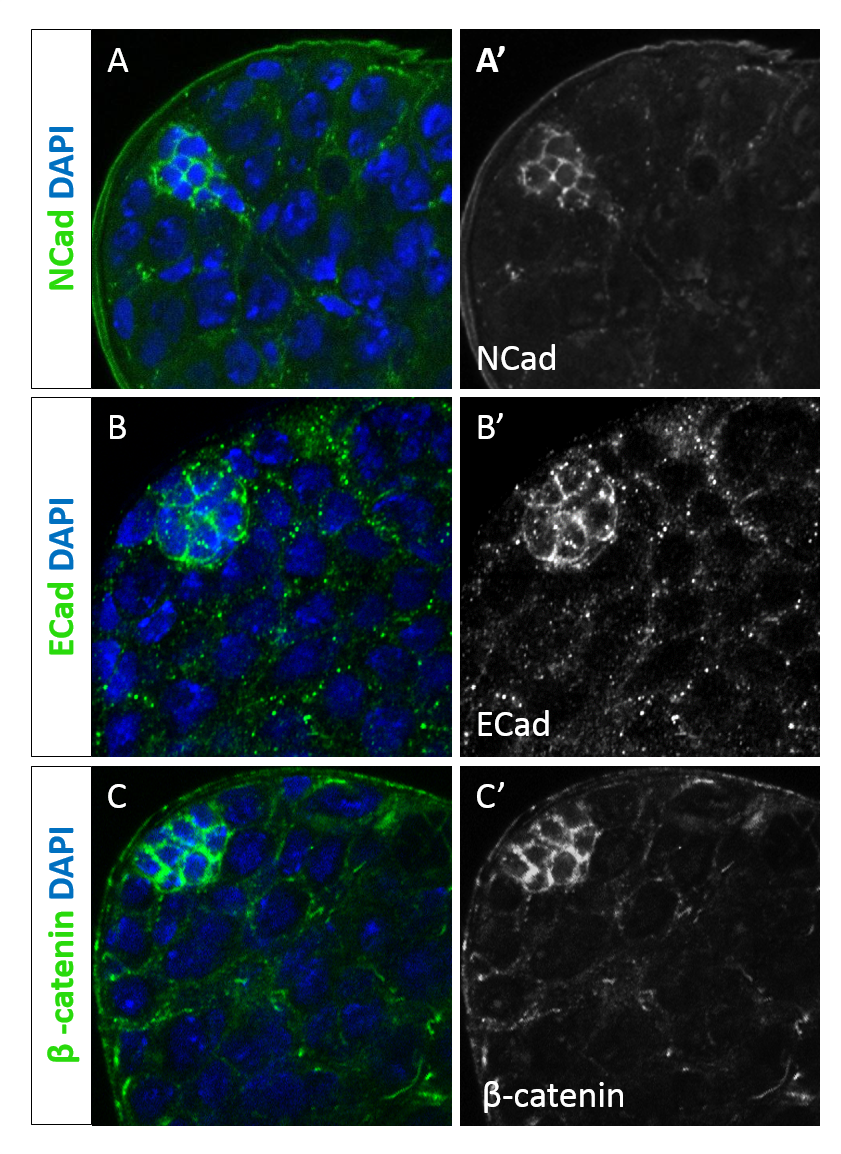

Supplement: Figure S4 — Adhesion complex factors are expressed in the testis and enriched in the niche. (A–C) Confocal sections of adult testes stained with DAPI (blue) and (A) anti-N-cadherin (green), (B) anti-E-cadherin (green) or (C) anti- β-catenin (green). N-cadherin, E-cadherin and β-catenin have similar hub enriched expression patterns. (A′, B′ and C′ show green channel alone.) (TIF) [file pgen.1004713.s004.tif]

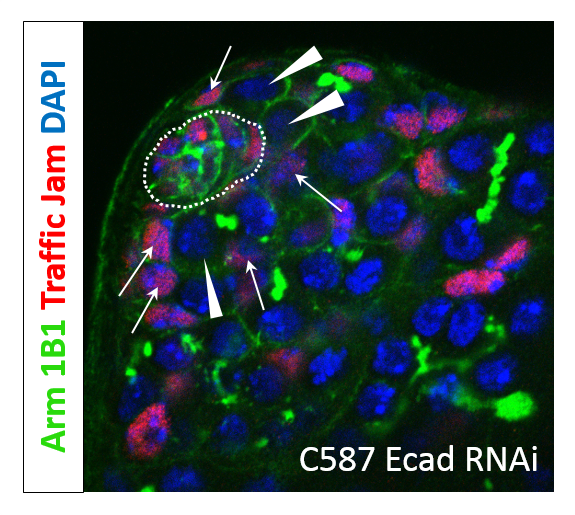

Supplement: Figure S5 — Knockdown of ECad in the entire CySC population does not lead to rapid CySC loss. Confocal section of an adult testis with traffic jam staining the hub and cyst lineage (red), Armadillo and 1B1 staining hub cells and fusome respectively (green) and DAPI staining DNA (blue). When ECad RNAi is expressed in the CySC lineage for 7 days via the C587-Gal4 driver, both GSCs (arrowheads) and CySCs (arrows) remain in the niche and testes appear grossly normal. Differentiating cyst cells and spermatogonia (identified by elongated fusomes) are evident away from the hub. (TIF) [file pgen.1004713.s005.tif]

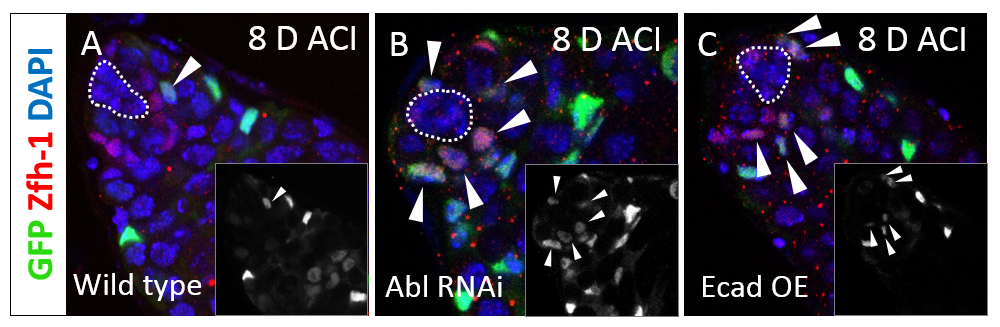

Supplement: Figure S6 — Clonal overexpression of Abl RNAi or ECad is sufficient to cause CySCs to outcompete their neighbors. (A–C) Confocal sections of testes with Zfh-1 staining CySCs and early cyst cell daughters (red). Positively marked mosaic clones are identified by presence of GFP (green). At 8 days ACI, (A) wild type mosaic testes contain a small number of marked CySCs per testis (arrowhead). Testes containing clones overexpressing (B) Abl RNAi or (C) ECad. Marked CySC outcompete their neighbors and testes contain many marked CySCs (arrowheads). Insets, GFP channel alone. (TIF) [file pgen.1004713.s006.tif]

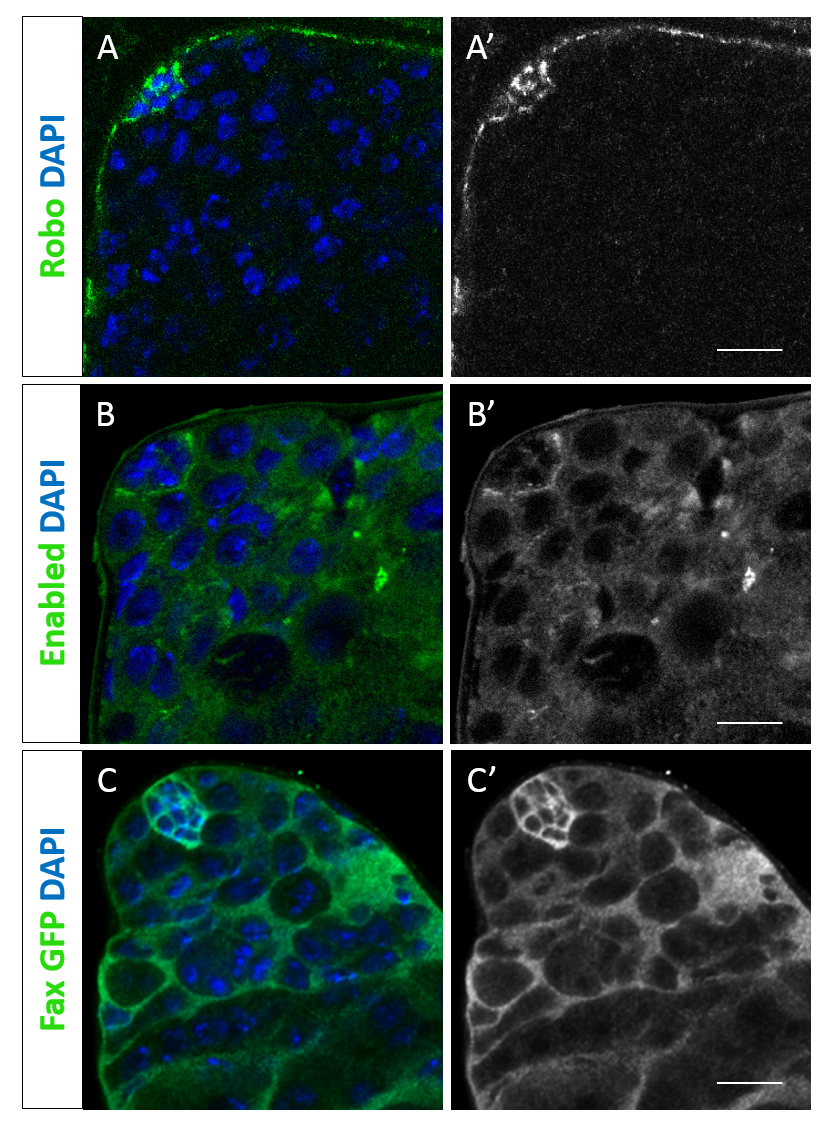

Supplement: Figure S7 — Factors involved in Abl kinase signaling are expressed in the testis apex. (A) Confocal section of an adult testis immunostained with DAPI (blue) and (A) anti-Robo (green) or (B) anti-Enabled (green). (A) Robo is expressed at hub-stem cell contacts. (A′) Robo staining alone (B) Enabled is expressed around hub cells and at hub-stem cell contacts. (B′) Enabled staining alone. (C) Confocal section of an adult testis expressing a Fax-GFP protein fusion stained with anti-GFP (green) and DAPI (blue). Fax is enriched in the hub and CySC lineage. (C′) GFP staining alone. Scale bars = 10 µm. (TIF) [file pgen.1004713.s007.tif]

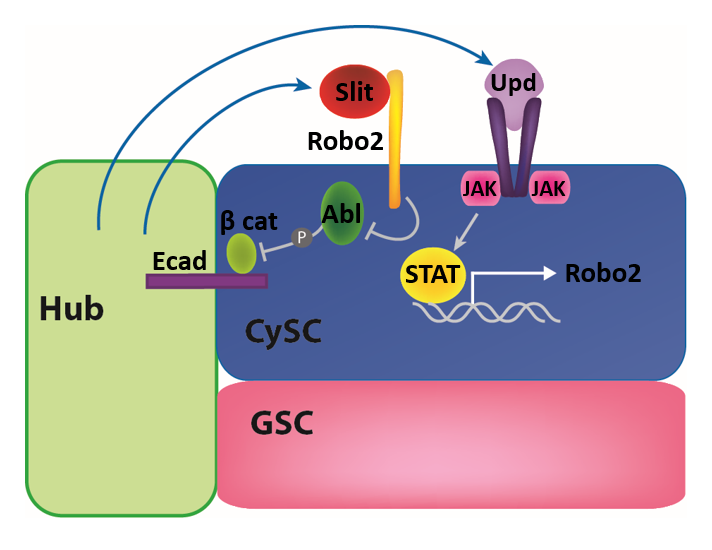

Supplement: Figure S8 — Model of JAK-STAT and Slit-Robo signaling interacting to control adhesion levels in the Drosophila testis niche. The axon guidance receptor Robo2 (orange) is required to regulate the level of adhesion in CySCs. The Robo2 ligand Slit (red) is expressed in the hub. Robo2 genetically interacts with Abl (green), antagonizing Abl activity to modulate adhesion levels. Abl can phosphorylate β-cat (light green), leading to the destabilization of the β-cat/ECad adherens junction complex and a decrease in cell adhesion levels. Robo2 transcription is controlled by the transcription factor Stat92E (yellow). Stat92E is activated by the Upd ligand (light purple), which is secreted from the hub and activates JAK-STAT signaling. Signaling in GSCs is not shown. (TIF) [file pgen.1004713.s008.tif]
