# Supplementary material for: Coordinate Regulation of Stem Cell Competition by Slit-Robo and JAK-STAT Signaling in the Drosophila Testis
Source: PLoS Genet. 2014 Nov 6;10(11):e1004713. doi: 10.1371/journal.pgen.1004713 (PMC4222695; doi:10.1371/journal.pgen.1004713)
Supplement: Table S1 — Robo2 is not required cell-autonomously for GSC maintenance in the Drosophila testis. (DOCX) [file pgen.1004713.s009.docx]

**Table S1 -** Robo2 is not required cell-autonomously for GSC maintenance in the *Drosophila* testis.

| **Genotype** | **2 days ACI** | **4 days ACI** | **8 days ACI** | **12 days ACI** |
| --- | --- | --- | --- | --- |
|  | **Testes with GSC Clones^a^** | | | |
| **Wild type clones** | 7/23  (30.4) | 8/22  (36.4) | 10/21  (47.6) | 5/19  (26.3) |
| **Robo2^1^ clones** | 8/25  (32.0) | 7/23  (30.4) | 10/25  (40.0) | 4/12  (33.3) |
| **Robo2^8^ clones** | 4/24  (16.7) | 9/24  (37.5) | 7/27  (25.9) | 7/26  (26.9) |

^a^ Testes with GSC clones = testes with GFP^-^, Vasa^+^ cells contacting the hub / total testes scored (percentage)

ACI = After Clone Induction
