# Supplementary material for: Coordinate Regulation of Stem Cell Competition by Slit-Robo and JAK-STAT Signaling in the Drosophila Testis
Source: PLoS Genet. 2014 Nov 6;10(11):e1004713. doi: 10.1371/journal.pgen.1004713 (PMC4222695; doi:10.1371/journal.pgen.1004713)
Supplement: Table S2 — Ncad is not required to maintain CySC clones in the Drosophila testis. (DOCX) [file pgen.1004713.s010.docx]

**Table S2** - Ncad is not required to maintain CySC clones in the *Drosophila* testis

| **Genotype** | **2 days ACI** | **4 days ACI** | **6 days ACI** | **10 days ACI** |
| --- | --- | --- | --- | --- |
|  | **Testes with CySC Clones^a^** | | | |
| **Wild type clones** | 13/31  (41.9) | 18/26  (69.2) | 20/46  (43.4) | 17/40  (42.5) |
| **Ncad^M19^ clones** | 4/22  (18.2) | 15/34  (44.1) | 11/38  (28.9) | 10/41  (24.4) |
| **Ncad^405^ clones** | 7/24  (29.2) | 9/17  (52.9) | 20/46  (43.4) | 13/42  (31.0) |

^a^ Testes with CySC clones = testes with GFP^+^, Zfh-1^+^ cells / total testes scored (percentage)

**^a^** Testes with CySC clones = testes with GFP^+^, Zfh-1^+^ cells/ total testes scored (percentage)

ACI = After Clone Induction

ACI = After Clone Induction
