# Supplementary material for: Coordinate Regulation of Stem Cell Competition by Slit-Robo and JAK-STAT Signaling in the Drosophila Testis
Source: PLoS Genet. 2014 Nov 6;10(11):e1004713. doi: 10.1371/journal.pgen.1004713 (PMC4222695; doi:10.1371/journal.pgen.1004713)
Supplement: Table S3 — Members of the Slit-Robo signaling pathway are expressed in stem cell enriched testes. (DOCX) [file pgen.1004713.s011.docx]

**Table S3 -** Members of the Slit-Robo signaling pathway are expressed in stem cell enriched testes.

| **Gene** | **Stem cell enriched (*bam*) testes RPKM^a^ value** | **Wildtype testes RPKM value** | **Fold Enrichment in *bam* testes** |
| --- | --- | --- | --- |
| **Robo** | 23.25 | 15.60 | 1.49 |
| **robo2/lea** | 10.51 | 1.85 | 5.69 |
| **Sli** | 22.75 | 7.85 | 2.90 |
| **robo3** | 0 | 1.10 | 0 |
| **Abl** | 40.74 | 20.30 | 2.01 |
| **Trio** | 36.72 | 11.51 | 3.19 |
| **Fax** | 164.82 | 76.69 | 2.15 |
| **Ena** | 45.16 | 6.90 | 6.54 |
| **NetA** | 14.85 | 1.38 | 10.78 |
| **NetB** | 3.09 | 1.14 | 2.71 |
| **Fra** | 50.42 | 18.89 | 2.67 |

^a^ Analysis of publically available RNA-seq data from Gan et al. 2010 comparing wildtype testes and testes enriched for stem cells by ectopic expression of bag-of-marbles (*bam*).

RPKM= Reads per kilobase per million
