# Supplementary material for: Coordinate Regulation of Stem Cell Competition by Slit-Robo and JAK-STAT Signaling in the Drosophila Testis
Source: PLoS Genet. 2014 Nov 6;10(11):e1004713. doi: 10.1371/journal.pgen.1004713 (PMC4222695; doi:10.1371/journal.pgen.1004713)
Supplement: Table S4 — CySCs lacking Robo are rapidly lost from the Drosophila testis niche. (DOCX) [file pgen.1004713.s012.docx]

**Table S4-** CySCs lacking Robo are rapidly lost from the *Drosophila* testis niche

| **Genotype** | **2 days ACI** | **4 days ACI** | **8 days ACI** | **12 days ACI** |
| --- | --- | --- | --- | --- |
|  | **Testes with CySC Clones^a^** | | | |
| **Wild type clones** | 16/31  (51.6) | 9/25  (36.0) | 10/25  (40.0) | 4/25  (16.0) |
| **Robo^1^ clones** | 0/35^***,b^  (0.0) | 0/27^***,b^  (0.0) | 0/21^***,b^  (0.0) | 0/26  (0.0) |
| **Robo^2^ clones** | 1/33^***,b^  (3.0) | 1/25^***,b^  (4.0) | 1/29^**,b^  (3.4) | 0/25  (0.0) |

**^a^** Testes with CySC clones = testes with GFP^+^, Zfh-1^+^ cells/ total testes scored (percentage)

^b^ P value vs wild type clones

ACI = After Clone Induction

** = P value<.01

*** = P value<.001
