# Supplementary material for: Coordinate Regulation of Stem Cell Competition by Slit-Robo and JAK-STAT Signaling in the Drosophila Testis
Source: PLoS Genet. 2014 Nov 6;10(11):e1004713. doi: 10.1371/journal.pgen.1004713 (PMC4222695; doi:10.1371/journal.pgen.1004713)
Supplement: Table S5 — Stat92E null clones are temporarily rescued by overexpression of Robo2. (DOCX) [file pgen.1004713.s013.docx]

**Table S5 –** *Stat92E* null clones are temporarily rescued by overexpression of Robo2

|  | **Stat^06346^ Control Clones** | **Stat^06346^ Clones overexpressing Robo2** |  |
| --- | --- | --- | --- |
| **Hours ACI** | **CySC Clones Per Testis ^a^** | | **P value ^b^** |
| **48** | 0.19 ± .08 | 0.55 ± .15 | .037 |
| **60** | 0.22 ± .08 | 0.35 ± .11 | .34 0 |

**^a^** CySC Clones Per Testis= Number of GFP^+^, Zfh1^+^ clones / total number of testes scored ± SEM

ACI = After Clone Induction.
