# Supplementary material for: Coordinate Regulation of Stem Cell Competition by Slit-Robo and JAK-STAT Signaling in the Drosophila Testis
Source: PLoS Genet. 2014 Nov 6;10(11):e1004713. doi: 10.1371/journal.pgen.1004713 (PMC4222695; doi:10.1371/journal.pgen.1004713)
Supplement: Table S6 — Full genotypes for mosaic analysis. (DOCX) [file pgen.1004713.s014.docx]

**Table S6** - Full genotypes for mosaic analysis.

| Wild Type Negative Clones (Robo2) | *w/Y ; Ubi-GFP.nls, FRT40A/ FRT40A ; MKRS, hsFLP/ +* |
| --- | --- |
| Robo2 Negative Clones | *w/Y ; Ubi-GFP.nls, FRT40A/ Robo2^1^* or *Robo2 ^8^, FRT40A ;MKRS, hsFLP/ +* |
| Wild Type Clones (Robo2 and Ncad) | *hs-FLP, tub-Gal4, UAS –GFP.nls/Y; FRT40A, tub-Gal80/ Frt40A* |
| Robo2 MARCM Clones | *hs-FLP, tub-Gal4, UAS –GFP.nls/Y; FRT40A, tub-Gal80/ Robo2^1^,Frt40A* |
| Ncad MARCM Clones | *hs-FLP, tub-Gal4, UAS –GFP.nls/Y; FRT40A, tub-Gal80/ Ncad^M19^*or Ncad*^Omb405^, Frt 40A* |
| Wild Type Clones (Abl) | *hs-FLP, tub-Gal4,UAS-CD8-GFP /Y; + ; tub-Gal80, FRT2A/ FRT2A* |
| Abl MARCM Clones | *hs-FLP, tub-Gal4,UAS-CD8-GFP/Y; + ; tub-Gal80, FRT2A/ FRT2A, Abl^1^* or *Abl^4^* |
| Abl overexpressing Abl | *hs-FLP, tub-Gal4,UAS-CD8-GFP/Y;+/ UAS-Abl; tub-Gal80, FRT2A/ FRT2A, Abl^4^* |
| Abl overexpressing Abl^KinaseDead^ | *hs-FLP, tub-Gal4,UAS-CD8-GFP/Y;+/ UAS-Abl^KinaseDead^; tub-Gal80, FRT2A/ FRT2A, Abl^4^* |
| Robo2 overexpressing Ecad | *hs-FLP, tub-Gal4, UAS –GFP.nls/Y; FRT40A, tub-Gal80/ Robo2^1^,Frt40A*;*UAS-Shg/+* |
| Robo2 with Abl RNAi | *hs-FLP, tub-Gal4, UAS –GFP.nls/Y; FRT40A, tub-Gal80/ Robo2^1^,Frt40A*; *UAS- Abl RNAi/+* |
| Abl with Ecad RNAi | *hs-FLP, tub-Gal4,UAS-CD8-GFP/Y;+/ UAS-EcadRNAi; tub-Gal80, FRT2A/ FRT2A, Abl^4^* |
| Abl with β-cat RNAi | *hs-FLP, tub-Gal4,UAS-CD8-GFP/Y;+/ UAS- β-catRNAi; tub-Gal80, FRT2A/ FRT2A, Abl^4^* |
| Wildtype overexpressing Ecad | *hs-FLP, tub-Gal4, UAS –GFP.nls/Y; FRT40A, tub-Gal80/ Frt40A*;*UAS-Shg/+* |
| Wildtype with Abl RNAi | *hs-FLP, tub-Gal4, UAS –GFP.nls/Y; FRT40A, tub-Gal80/ Frt40A*; *UAS-Abl RNAi/+* |
| Wildtype with Ecad RNAi | *hs-FLP, tub-Gal4,UAS-CD8-GFP/Y;+/ UAS- EcadRNAi; tub-Gal80, FRT2A/ FRT2A* |
| Wildtype with β-cat RNAi | *hs-FLP, tub-Gal4,UAS-CD8-GFP/Y;+/ UAS-β-catRNAi; tub-Gal80, FRT2A/ FRT2A* |
| β-cat Phosphorylation Variants with Robo2 RNAi | *hs-Flp/Y; Tub>CD2>Gal4, UAS-GFP/UAS-* β-cat *(WT, Y667F or Y667E); UAS-Robo2-RNAi* |
| Wild type clones (Robo) | *w/Y ; Ubi-GFP.nls, FRT42B/ FRT42B ; MKRS, hsFLP/ +* |
| Robo Negative Clones | *w/Y ; Ubi-GFP.nls, FRT42B/ Robo^1^* or *Robo2^8^, FRT42B ;MKRS, hsFLP/ +* |
| Stat92E MARCM Clones | *hs-FLP, UAS-CD8-GFP/Y; +; tub-GAL4, FRT82B, tub-GAL80/Frt 82B Stat92E^06346^* |
| Stat92E overexpressing Robo2 | *hs-FLP, UAS-CD8-GFP/Y; +/ UAS-Robo2-HA; tub-GAL4, FRT82B, tub-GAL80/ Frt 82B* |
