# Supplementary material for: Coordinate Regulation of Stem Cell Competition by Slit-Robo and JAK-STAT Signaling in the Drosophila Testis
Source: PLoS Genet. 2014 Nov 6;10(11):e1004713. doi: 10.1371/journal.pgen.1004713 (PMC4222695; doi:10.1371/journal.pgen.1004713)
Supplement: Protocol S1 — Detailed methods, antibody and fly stock information. (DOCX) [file pgen.1004713.s015.docx]

**Supplemental Methods**

**Antibodies and Flies**

Additional antibodies used were rat anti-DN cadherin (DSHB, 1:20), rat anti-DE cadherin (DSHB, 1:10), mouse anti-armadillo (DSHB, 1:50), mouse anti-Robo (DSHB, 1:10) and mouse anti-Enabled (DSHB, 1:20). For Robo staining, testes were dissected onto slides and fixed with methanol and acetone as described in Bonaccorsi et al. [86]. Additional fly lines were y,*w; UAS-sli.B/TM3* (Bloomington, 6472) *w; Ubi-GAL4/CyO* (Bloomington, 32551), *Fax-GFP^CC01359^* (A. Spradling, [31]) *hs-FLP, UAS-CD8-GFP; + ; tub-GAL4, FRT82B, tub-GAL80* (B. Ohlstein), *Frt82B, Stat92E^06346^* [18], *w, UAS-Robo2-HA/CyO* (G. Bashaw), and *UAS-Arm-WT*, *UAS-Arm Y667F* and *UAS-Arm667E* (all from J. Zallen, published in Tamada et al. [52].) Genotypes for all mosaic analysis experiments can be found in Table S10.

**Scoring and Quantifying GSCs and CySCs**

All clonal experiments were stained with GFP (to identify clones), Zfh-1 (to mark CySCs and early daughters), and DAPI to mark DNA. GSCs were identified as DAPI-positive, Zfh-1 negative cells contacting the hub. CySCs and their immediate daughters were identified as cells with medium to strong Zfh-1 levels according to the rainbow indicator provided in the Zeiss Pascal software. The number of CySCs per testis was determined using serial confocal sections of the testis apex as in Issigonis et al. [18]. Since Zfh-1 marks both CySCs and their immediate daughter cyst cells, an approximate CySC count was determined by dividing the total number of Zfh-1-positive cells by 2. Mitotic Zfh-1 positive cells were identified by double staining with Zfh-1 and the mitotic marker, phospho-Histone H3. To quantify dying cells, 0-5 day old flies were heat shocked (37˚C) for 45 minutes two times per day with one hour recovery (25˚C) on two consecutive days to induce *robo2* or wild type control clones and dissected 48 hours after initial heat shock. Apoptosis was detected by the indirect TUNEL method using an Apoptag Red In Situ Apoptosis Detection Kit (Millipore). All Apoptag-positive cells within two cell nuclei of the hub (as scored by DAPI) were counted as probable dying stem cells. Statistical analysis of stem cell number, mitotic index, and apoptotic cell number was performed using the GraphPad online program. P-values were obtained between two groups using Fisher exact or Student’s T test. Pixel intensity calculations were done on projected Z-stack confocal images using the histogram analysis tool on Image J. All intensity measurement were normalized to DAPI intensity.

**Additional Information on Stocks and Reagents**

Polyclonal rabbit anti-Robo2 was originally generated and published by Rajagopalan et al.[23]. Monoclonal mouse anti-Slit staining was originally generated and published by Rothberg et al. [87]. *robo2^1^* and *robo2^8^* alleles were originally generated and published by Rajagopalan et al. [23]. Polyclonal guinea pig anti-Zfh-1 was first published in the testis by Sheng et al. [73]. Polyclonal guinea pig anti-traffic jam was generated and staining was originally published in male and female gonads by Li et al. [88]. The *Ncad^M19^* and *Ncad^40^*^5^ alleles were previously characterized [34,89] and mosaic clones stocks for both alleles were generated by Lee et al. [34]. The *Abl^1^* and *Abl^4^* alleles were orignally generated and published by the Hoffmann laboratory [90,91]. *Abl:GFP* was generated by the Peifer lab by fusing the open reading frame of *Abl* plus 2 kilobases of upstream regulatory elements to GFP and randomly inserting the contruct into *Drosophila* to create a transgenic line. This fly line was originally described in embryonic epithelial tissue [45]. Monoclonal mouse anti-Robo was originally generated and published by Kidd et al. [92]. Robo^1^ and Robo^2^ alleles were originally generated and published but Seeger et al. [55] and are available from Bloomington (Stocks 8755 and 8756). Monoclonal mouse anti-Enabled staining was originally generated and published by Bashaw et al. [29]and staining was later published in the testis [75]. Induction of JAK-STAT signaling using *HS-Upd* (generated by the Harrison lab) was first described by McGregor et al. [93]. The *stat92E* null allele *stat92E^06346^* was identified and characterized by Hou et al. [94], and the temperature senstitive *Stat92E^Frankenstein^* allele was identified and characterized by Baska et al. [95].

86. Bonaccorsi S, Giansanti MG, Cenci G, Gatti M (2011) Methanol-acetone fixation of Drosophila testes. Cold Spring Harb Protoc 2011: 1270-1272.

87. Rothberg JM, Jacobs JR, Goodman CS, Artavanis-Tsakonas S (1990) slit: an extracellular protein necessary for development of midline glia and commissural axon pathways contains both EGF and LRR domains. Genes Dev 4: 2169-2187.

88. Li MA, Alls JD, Avancini RM, Koo K, Godt D (2003) The large Maf factor Traffic Jam controls gonad morphogenesis in Drosophila. Nat Cell Biol 5: 994-1000.

89. Iwai Y, Usui T, Hirano S, Steward R, Takeichi M, et al. (1997) Axon patterning requires DN-cadherin, a novel neuronal adhesion receptor, in the Drosophila embryonic CNS. Neuron 19: 77-89.

90. Gertler FB, Bennett RL, Clark MJ, Hoffmann FM (1989) Drosophila abl tyrosine kinase in embryonic CNS axons: a role in axonogenesis is revealed through dosage-sensitive interactions with disabled. Cell 58: 103-113.

91. Bennett RL, Hoffmann FM (1992) Increased levels of the Drosophila Abelson tyrosine kinase in nerves and muscles: subcellular localization and mutant phenotypes imply a role in cell-cell interactions. Development 116: 953-966.

92. Kidd T, Brose K, Mitchell KJ, Fetter RD, Tessier-Lavigne M, et al. (1998) Roundabout controls axon crossing of the CNS midline and defines a novel subfamily of evolutionarily conserved guidance receptors. Cell 92: 205-215.

93. McGregor JR, Xi R, Harrison DA (2002) JAK signaling is somatically required for follicle cell differentiation in Drosophila. Development 129: 705-717.

94. Hou XS, Melnick MB, Perrimon N (1996) Marelle acts downstream of the Drosophila HOP/JAK kinase and encodes a protein similar to the mammalian STATs. Cell 84: 411-419.

95. Baksa K, Parke T, Dobens LL, Dearolf CR (2002) The Drosophila STAT protein, stat92E, regulates follicle cell differentiation during oogenesis. Dev Biol 243: 166-175.
